# Supplementary material for: Urbanization strengthens vertical stratification of ant nutrient preferences in a temperate forest ecosystem
Source: PLoS One. 2025 Mar 28;20(3):e0320380. doi: 10.1371/journal.pone.0320380 (PMC11952225; doi:10.1371/journal.pone.0320380)
Supplement: S1 File — Document containing 3 supplementary texts, 7 supplementary tables, 5 supplementary figures, and references. S1 Text. Detailed bait design and methods. S2 Text. Description of Tapinoma cf. sessile. S3 Text. Analysis of variation in bait heights. S1 Table. Nutritional content of tuna and jam baits. S2 Table. Complete list of taxonomic keys used in this study. S3 Table. Substitute species biomass values. List of species for whom we could not calculate average individual weights and the species’ values used as their substitute weights. S4 Table. Occurrence of ant species in arboreal and terrestrial baits across seven forest and seven urban habitats in central North Carolina, USA. S5 Table. Pairwise comparisons of changes in ant community composition. S6 Table. Contrasts of the three-way interaction between stratum, habitat, and nutrient from the model predicting ant biomass. S7 Table. Species specific biomass estimates and model results for four urban biomass-dominant species. S1 Fig. Bait height varied with habitat and tree species. S2 Fig. Ant diversity at baits did not significantly differ between habitats and strata. S3 Fig. Changes in ant communities between the ground and canopy were due to species turnover. S4 Fig. Changes in ant communities between the forest and urban habitats were due to species turnover. S5 Fig. Ant biomass was significantly greater on the ground than in the canopy. (DOCX) [file pone.0320380.s001.docx]

*PLOS One*: Research Article

**Supporting information** for Urbanization strengthens vertical stratification of ant nutrient preferences in a temperate forest ecosystem

Michelle Kirchner, Lucie Ciccone, Clyde Sorenson, Elsa Youngsteadt

**Supporting Text**

**Text S1. Detailed bait design and methods**.

Each bait station consisted of a 15cm pine dowel (2.54cm diameter) bearing a #8 eye screw in its center. On each end of the dowel, we attached a 1in diameter polystyrene snap-on lid with a #8 screw and washer. For the bait vials, we used 9-dram styrene tubes (BioQuip, 1 x 2.75in) and drilled 4 entry holes into the lower half of the tube with a 2mm drill bit. Holes of this diameter allowed even the largest ants in our area to enter the bait vials, while excluding many other animals. Bycatch was infrequent and the commonest organisms other than ants were *Vespula* wasps, flies in family Calliphoridae, and bees in subfamily Apinae, all of which were easily released prior to collection.

To place arboreal baits in the canopy, we installed parachute cord over a sturdy branch with a BigShot^TM^ slingshot at least three days before sampling. During sampling, we attached the bait station to a loop in the cord with a small carabiner and hoisted it until flush with the anchoring branch, such that both bait vials were in contact with the tree’s trunk or a main branch. During collection, we lowered the bait station with the cord, taking care not to knock it against the tree. Foraging ants usually remained undisturbed if the bait station was lowered steadily. We collected all ants in each bait vial by snapping the tube off the attached lid into a 4oz Whirl-Pak® of 70% ethanol.

**Text S2. Description of *Tapinoma cf. sessile*.**

In more than half of bait samples containing what appeared to be *Tapinoma sessile*, the ants keyed out to this species, and while they match the species description morphologically, they differ in coloration. *Tapinoma sessile* is concolorous light to dark brown, while these specimens are consistently bicolored, such that the metasoma is medium to dark brown and the head and mesosoma are light yellowish-brown. It has been suggested that *T. sessile* in North America is a species complex, rather than a single species (Fisher and Cover 2007), and the bicolored *Tapinoma schreiberi* was split from *Tapinoma sessile* based on subtle morphological differences, coloration, and behavior (Hamm 2010). We therefore classified these bicolored specimens as *Tapinoma cf. sessile* based on coloration and expert consultation (J. Trager, personal communication). Vouchers are deposited in the North Carolina State University Insect Museum.

**Text S3. Analysis of variation in bait heights.**

In each tree, we placed two baits, each on a relatively large branch in the canopy. Bait placement was affected by canopy structure, as well as canopy access. Particularly in the urban sites, canopy access was often limited by powerlines, busy roadways, pedestrians/residents, nearby windows, and parked cars. This resulted in a large range of bait heights (2.1–31.1m). To understand how bait height variation may have affected our results, we constructed a linear mixed model using *lme4* 1.1-31. We modeled bait height as a function of habitat type, tree species, and their interaction with random effects of site and tree. We checked model fit with *DHARMa* 0.4.6 and tested significance using Type II Wald χ^2^ tests in *car* 3.1-1.

Baits in the forest were, on average, 6.6m higher than baits in urban trees (habitat: χ^2^=31.6, df=1, p<0.001), and baits in white oak trees were 5.3m higher than those in red maples (tree species: χ^2^=52.8, df=1, p<0.001) (Fig. SXX). However, there was no interaction between habitat and tree species (habitat*tree species: χ^2^=5.5, df=1, p=0.019)—that is, the height differences between the tree species were equal in both habitats. Therefore, we don’t expect bait height differences to affect our focal comparisons between forest and urban habitats.

Because baits in the forest were higher, it is possible that they were less accessible to ground foragers, and/or the ground was less accessible to canopy foragers, than in the urban habitat. If this were driving our results, we would expect to see less cross-stratum foraging in the forest than in urban habitats, but despite the height difference between habitats, 50% of species, representing 98% of ant biomass, foraged across strata in both urban and forest sites. Additionally, if our results were due to ground-dwelling ants in urban sites having easier access to arboreal baits than those in forest sites, we would expect to see increased ant biomass in both urban arboreal protein and urban arboreal carbohydrate baits when compared to forest biomass estimates. Instead, absolute biomass estimates in arboreal carbohydrates were nearly equal in forest and urban trees—ant biomass only increased in urban arboreal protein baits. Thus, we are reasonably confident that differences bait height between habitats did not contribute to our findings.

**Supporting Tables**

**Table S1. Nutritional content of tuna and jam baits.**

|  | **StarKist^®^ chunk light tuna in water** | **Smucker’s^®^  strawberry preserves** |
| --- | --- | --- |
| **Average bait size (N=6)** | 8.93g | 13.35g |
| **Calories** | 7.03 | 33.38 |
| **Total Fat** | 0.04g | 0g |
| **Cholesterol** | 3.56mg | 0mg |
| **Sodium** | 28.45mg | 0mg |
| **Total Carbohydrate** | 0g | 8.68g |
| **Protein** | 1.58g | 0g |
| **Vitamin D** | 0.17μg | 0μg |
| **Iron** | 0.13mg | 0mg |
| **Potassium** | 15.81mg | 0mg |
| **Also contains** | Niacin, Vitamin B6,  Vitamin B12, Selenium | -- |
| **Ingredients** | Light Tuna, Water,  Vegetable Broth, Salt | Strawberries, High Fructose Corn Syrup, Corn Syrup, Sugar,  Fruit Pectin, Citric Acid |

**Table S2. Complete list of taxonomic keys used in this study.**

| **Genus** | **Taxonomic References** |
| --- | --- |
| *Aphaenogaster* | DeMarco 2015 |
| *Brachymyrmex* | Ortiz-Sepulveda et al. 2019 |
| *Camponotus* | MacGown 2022 |
| *Colobopsis* | MacGown 2022 |
| *Crematogaster* | Ward and Blaimer 2022; MacGown 2022 |
| *Formica* | Creighton 1950; Francoeur 1973; Trager, MacGown, and Trager 2007 |
| *Lasius* | AntWiki 2018 |
| *Linepithema* | Fisher and Cover 2007 |
| *Monomorium* | AntWiki 2019 |
| *Nylanderia* | Kallal and LaPolla 2012; MacGown 2022 |
| *Pheidole* | Gregg 1958; MacGown 2022 |
| *Pseudomyrmex* | AntWiki 2014a |
| *Solenopsis* | Pacheco and Mackay 2013; MacGown 2022 |
| *Tapinoma* | AntWiki 2014b |
| *Temnothorax* | MacGown 2022 |
| *Tetramorium* | AntWiki 2017 |

**Table S3. Substitute species biomass values.** List of species for whom we could not calculate average individual weights and the species’ values used as their substitute weights.

| **Species** | **Substitute species** | **Substitute weight [mg]** |
| --- | --- | --- |
| *Camponotus americanus* | *Camponotus castaneus* | 7.67 |
| *Camponotus caryae* | *Camponotus nearcticus* | 3.01 |
| *Temnothorax pergandei* | *Temnothorax schaumii* | 0.12 |

**Table S4. Occurrence of ant species in arboreal and terrestrial baits across seven forest and seven urban habitats in central North Carolina, USA.** Values are the number of sites within a given habitat, stratum, and nutrient from which the ant species was collected (max=7). Values in parentheses are the number of trees within a given habitat, stratum, and nutrient from which the ant species was collected (forest max=42, urban max=41). Bold numbers to the left of the species name correspond to Figure 4. Species lacking an authority are morphospecies or species complexes. Nutrient abbreviations: C = carbohydrate, P = protein.

|  |  |  | **Forest** | | | | **Urban** | | | |
| --- | --- | --- | --- | --- | --- | --- | --- | --- | --- | --- |
|  |  |  | **Arboreal** | | **Terrestrial** | | **Arboreal** | | **Terrestrial** | |
| **Subfamily** |  | **Species** | **C** | **P** | **C** | **P** | **C** | **P** | **C** | **P** |
| **Myrmicinae** | **1** | *Aphaenogaster lamellidens* Mayr 1886 | -- | -- | 2 (2) | -- | -- | -- | -- | -- |
|  | **2** | *Aphaenogaster mariae* Forel 1886 | 1 (1) | 1 (1) | -- | -- | -- | -- | -- | -- |
|  | **3** | *Aphaenogaster rudis s.l.* | -- | -- | 4 (9) | 3 (3) | -- | -- | 2 (2) | -- |
|  | **4** | *Aphaenogaster tennesseensis* (Mayr 1862) | 1 (1) | -- | 1 (1) | -- | -- | -- | -- | -- |
|  | **5** | *Aphaenogaster treatae* Forel 1886 | -- | -- | -- | -- | -- | -- | 2 (2) | 1 (1) |
| **Formicinae** | **6** | *Brachymyrmex patagonicus* Mayr 1868 | -- | -- | -- | -- | 4 (5) | 2 (3) | 5 (13) | 3 (5) |
| **Ponerinae** | **7** | *Brachyponera chinensis* (Emery 1895) | 1 (2) | -- | 5 (7) | 5 (19) | -- | -- | 2 (2) | 2 (2) |
| **Table S4** *cont.* |  |  | **Forest** | | | | **Urban** | | | |
|  |  |  | **Arboreal** | | **Terrestrial** | | **Arboreal** | | **Terrestrial** | |
| **Subfamily** |  | **Species** | **C** | **P** | **C** | **P** | **C** | **P** | **C** | **P** |
| **Formicinae** | **8** | *Camponotus americanus* Mayr 1862 | 2 (2) | 1 (1) | 1 (1) | 1 (1) | -- | -- | -- | -- |
|  | **9** | *Camponotus caryae* (Fitch 1855) | -- | -- | -- | -- | 2 (2) | 3 (4) | -- | -- |
|  | **10** | *Camponotus castaneus* (Latreille 1802) | -- | 1 (1) | 4 (5) | 4 (4) | -- | -- | -- | -- |
|  | **11** | *Camponotus chromaiodes* Bolton 1995 | 2 (4) | 2 (4) | 5 (15) | 6 (14) | 1 (1) | 2 (2) | 1 (1) | -- |
|  | **12** | *Camponotus nearcticus* Emery 1893 | 6 (15) | 6 (18) | 1 (1) | 1 (1) | 3 (5) | 5 (6) | -- | -- |
|  | **13** | *Camponotus pennsylvanicus* (De Geer 1773) | 4 (4) | 6 (6) | 3 (4) | 4 (8) | 3 (3) | 4 (5) | 3 (5) | 2 (3) |
|  | **14** | *Camponotus snellingi* Bolton 1995 | 2 (3) | -- | -- | -- | -- | -- | -- | -- |
|  | **15** | *Camponotus subbarbatus* Emery 1893 | -- | 1 (1) | 1 (1) | 1 (1) | -- | -- | -- | -- |
|  | **16** | *Colobopsis impressa* Roger 1863 | -- | 1 (1) | -- | -- | 2 (2) | 3 (3) | -- | -- |
|  | **17** | *Colobopsis mississippiensis* (Smith 1923) | -- | -- | -- | -- | -- | 1 (1) | -- | -- |
|  | **18** | *Colobopsis obliqua* (Smith 1930) | 1 (1) | -- | -- | -- | -- | 3 (3) | -- | 1 (1) |
| **Myrmicinae** | **19** | *Crematogaster ashmeadi* Mayr 1886 | 5 (7) | 7 (19) | -- | 1 (1) | 5 (8) | 7 (21) | 4 (5) | 5 (6) |
|  | **20** | *Crematogaster lineolata* (Say 1836) | -- | -- | 2 (2) | -- | -- | -- | -- | -- |
|  | **21** | *Crematogaster pilosa* (Emery 1895) | -- | -- | -- | -- | 1 (1) | 1 (1) | -- | 1 (1) |
| **Table S4** *cont.* |  |  | **Forest** | | | | **Urban** | | | |
|  |  |  | **Arboreal** | | **Terrestrial** | | **Arboreal** | | **Terrestrial** | |
| **Subfamily** |  | **Species** | **C** | **P** | **C** | **P** | **C** | **P** | **C** | **P** |
| **Myrmicinae** | **22** | *Crematogaster vermiculata* Emery 1895 | -- | -- | 1 (1) | -- | -- | -- | -- | -- |
| **Formicinae** | **23** | *Formica biophilica* Trager 2007 | -- | -- | -- | -- | 3 (3) | 2 (2) | 4 (8) | 2 (2) |
|  | **24** | *Formica pallidefulva* Latreille 1802 | 1 (1) | -- | 3 (9) | -- | 1 (1) | -- | 2 (3) | -- |
|  | **25** | *Formica subsericea* Say 1836 | 5 (7) | 3 (5) | 7 (17) | 6 (15) | 4 (10) | 3 (9) | 5 (11) | 4 (7) |
|  | **26** | *Lasius americanus* Emery 1893 | -- | -- | 1 (1) | 1 (1) | -- | -- | -- | -- |
|  | **27** | *Lasius neoniger* Emery 1893 | -- | -- | -- | -- | 1 (1) | -- | 4 (7) | 4 (4) |
| **Dolichoderinae** | **28** | *Linepithema humile* (Mayr 1868) | -- | -- | -- | -- | 1 (1) | -- | 1 (1) | 1 (1) |
| **Myrmicinae** | **29** | *Monomorium minimum* (Buckley 1867) | -- | -- | -- | -- | 4 (4) | 3 (4) | 7 (21) | 7 (16) |
| **Formicinae** | **30** | *Nylanderia concinna* (Trager 1984) | -- | -- | -- | -- | 2 (2) | -- | 6 (14) | 5 (7) |
|  | **31** | *Nylanderia faisonensis* (Forel 1922) | -- | -- | 4 (8) | 2 (2) | -- | -- | 5 (9) | 3 (4) |
|  | **32** | *Nylanderia flavipes* (Smith 1874) | -- | -- | -- | -- | -- | -- | 2 (2) | -- |
|  | **.** | *Nylanderia parvula* (Mayr 1870) | -- | -- | -- | -- | -- | -- | 1 (1) | 1 (1) |
|  | **34** | *Nylanderia vividula* (Nylander 1846)/ *Nylanderia terricola* (Buckley 1866)* | -- | -- | 1 (1) | 1 (1) | -- | -- | 6 (16) | 3 (4) |
| **Myrmicinae** | **35** | *Pheidole bicarinate* Mayr 1870 | -- | -- | -- | -- | -- | -- | 2 (3) | 1 (1) |
| **Table S4** *cont.* |  |  | **Forest** | | | | **Urban** | | | |
|  |  |  | **Arboreal** | | **Terrestrial** | | **Arboreal** | | **Terrestrial** | |
| **Subfamily** |  | **Species** | **C** | **P** | **C** | **P** | **C** | **P** | **C** | **P** |
| **Myrmicinae** | **36** | *Pheidole dentata* Mayr 1886 | -- | -- | -- | -- | -- | -- | 1 (2) | 1 (3) |
|  | **37** | *Pheidole tysoni* Forel 1901 | -- | -- | -- | -- | -- | -- | 1 (1) | 1 (1) |
| **Pseudomyrmecinae** | **38** | *Pseudomyrmex ejectus* (Smith 1858) | -- | -- | -- | -- | -- | 1 (1) | -- | -- |
| **Myrmicinae** | **39** | *Solenopsis invicta* Buren 1972 | 1 (1) | -- | 1 (1) | -- | 2 (2) | 1 (2) | 5 (13) | 5 (14) |
|  | **40** | *Solenopsis molesta s.l.* | -- | -- | -- | -- | 1 (1) | -- | 4 (6) | 4 (4) |
| **Dolichoderinae** | **41** | *Tapinoma cf. sessile* | -- | -- | -- | -- | 2 (2) | 2 (2) | 5 (8) | 3 (5) |
|  | **42** | *Tapinoma sessile* (Say 1836) | -- | -- | 1 (1) | -- | 3 (3) | 3 (3) | 4 (6) | 4 (5) |
| **Myrmicinae** | **43** | *Temnothorax bradleyi* (Wheeler 1913) | -- | -- | -- | -- | 2 (3) | -- | -- | -- |
|  | **44** | *Temnothorax curvispinosus* (Mayr 1866) | -- | 1 (1) | 3 (4) | 2 (4) | -- | -- | 1 (1) | 1 (1) |
|  | **45** | *Temnothorax pergandei* (Emery 1895) | -- | -- | -- | -- | -- | -- | 1 (1) | -- |
|  | **46** | *Temnothorax schaumii* (Roger 1863) | 3 (4) | 2 (2) | -- | -- | 3 (3) | 2 (2) | -- | 1 (1) |
|  | **47** | *Tetramorium immigrans* Santschi 1927 | -- | -- | -- | -- | -- | -- | 1 (2) | 1 (1) |

**Nylanderia vividula* and *N. terricola* workers cannot be reliably separated based on morphology, so we treated these two species as one for this study.

**Table S5. Pairwise comparisons of changes in ant community composition.** Pairwise comparisons of community median Jaccard distances between levels of the habitat type (forest/urban) and stratum (arboreal/terrestrial) interaction in PERMANOVA model assessing the changes in ant community composition. Asterisks indicate p < 0.05.

| **Group 1** | **Group 2** | **R^2^** | **F-value** | **Numerator  df** | **Denominator  df** | **p-value** |  |
| --- | --- | --- | --- | --- | --- | --- | --- |
| urban arboreal | urban terrestrial | 0.225 | 3.49 | 1 | 12 | 0.001 | ***** |
| urban arboreal | forest arboreal | 0.189 | 2.805 | 1 | 12 | 0.002 | ***** |
| urban arboreal | forest terrestrial | 0.328 | 5.845 | 1 | 12 | 0.001 | ***** |
| urban terrestrial | forest arboreal | 0.413 | 8.426 | 1 | 12 | 0.001 | ***** |
| urban terrestrial | forest terrestrial | 0.36 | 6.751 | 1 | 12 | 0.001 | ***** |
| forest arboreal | forest terrestrial | 0.289 | 4.877 | 1 | 12 | 0.001 | ***** |

**Table S6. Contrasts of the three-way interaction between stratum, habitat, and nutrient from the model predicting ant biomass.** Simple main effect contrasts of interaction between stratum (arboreal/terrestrial), habitat (forest/urban) and nutrient (carbohydrate/protein) from linear mixed model predicting log+1 transformed ant biomass. Asterisks indicate p < 0.05.

| **Site type** | **Stratum** | **Nutrient** | **Contrast** | **Ratio** | **Std. error** | **df** | **null** | **t ratio** | **p-value** |  |
| --- | --- | --- | --- | --- | --- | --- | --- | --- | --- | --- |
| forest | -- | C | arboreal / terrestrial | 0.306 | 0.0916 | 243 | 1 | -3.96 | <0.001 | ***** |
| forest | -- | P | arboreal / terrestrial | 0.4 | 0.12 | 243 | 1 | -3.06 | 0.009 | ***** |
| urban | -- | C | arboreal / terrestrial | 0.144 | 0.0434 | 243 | 1 | -6.42 | <0.001 | ***** |
| urban | -- | P | arboreal / terrestrial | 0.928 | 0.281 | 243 | 1 | -0.247 | 0.999 |  |
| forest | arboreal | -- | C / P | 0.873 | 0.261 | 243 | 1 | -0.453 | 0.985 |  |
| forest | terrestrial | -- | C / P | 1.14 | 0.341 | 243 | 1 | 0.443 | 0.986 |  |
| urban | arboreal | -- | C / P | 0.312 | 0.0944 | 243 | 1 | -3.85 | <0.001 | ***** |
| urban | terrestrial | -- | C / P | 2.02 | 0.61 | 243 | 1 | 2.32 | 0.082 |  |
| -- | arboreal | C | forest / urban | 1.21 | 0.549 | 26.5 | 1 | 0.427 | 0.976 |  |
| -- | terrestrial | C | forest / urban | 0.569 | 0.257 | 26.5 | 1 | -1.25 | 0.526 |  |
| -- | arboreal | P | forest / urban | 0.434 | 0.196 | 26.5 | 1 | -1.85 | 0.212 |  |
| -- | terrestrial | P | forest / urban | 1 | 0.455 | 26.5 | 1 | 0.0101 | 1 |  |

**Table S7. Species specific biomass estimates and model results for four urban biomass-dominant species.** Measured biomass estimates and Wald Type II (marginal) χ^2^ tests of the linear mixed models predicting log+1 transformed ant biomass as a function of stratum, site type, nutrient, and their interactions for the four urban biomass-dominant species from this study. Percents under species name represent the proportion of total urban biomass represented by the species. Df =1 for all model predictors. Values in bold indicate statistical significance at the 0.05 level.

|  | *Formica subsericea*  28.8% | | *Crematogaster ashmeadi*  16.2% | | *Camponotus pennsylvanicus*  12.8% | | *Solenopsis invicta*  10.1% | |
| --- | --- | --- | --- | --- | --- | --- | --- | --- |
| **Biomass** [mg] | Forest | Urban | Forest | Urban | Forest | Urban | Forest | Urban |
| Arboreal | 389.5 | 1141.6 | 243.3 | 1098.1 | 387.8 | 421.1 | 0.3 | 64.8 |
| Terrestrial | 2844.2 | 1239.9 | 0.2 | 247.2 | 801.7 | 635.4 | 0.3 | 773.8 |
| **Predictor** | χ**^2^** | **p(>**χ**^2^)** | χ**^2^** | **p(>**χ**^2^)** | χ**^2^** | **p(>**χ**^2^)** | χ**^2^** | **p(>χ^2^)** |
| Stratum:  *terrestrial/arboreal* | 8.3 | **0.004** | 3.6 | 0.057 | 0.1 | 0.777 | 3.4 | 0.067 |
| Habitat:  *forest/urban* | 0.2 | 0.668 | 10.4 | 0.001 | 0 | 0.911 | 1.5 | 0.217 |
| Nutrient:  *protein/carbohydrate* | 22.3 | **<0.001** | 177.2 | **<0.001** | 13.4 | **<0.001** | 0.1 | 0.720 |
| Stratum*Habitat | 0.4 | 0.549 | 0.6 | 0.429 | 1.9 | 0.169 | 0.4 | 0.510 |
| Stratum*Nutrient | 0 | 0.949 | 7.4 | **0.006** | 0.1 | 0.767 | 0.5 | 0.498 |
| Habitat*Nutrient | 1.1 | 0.295 | 12.4 | **<0.001** | 0.6 | 0.452 | 0.2 | 0.636 |
| Stratum*Habitat*Nutrient | 0.9 | 0.333 | 0.1 | 0.799 | 1.4 | 0.239 | 0.1 | 0.762 |

**Supporting Figures**


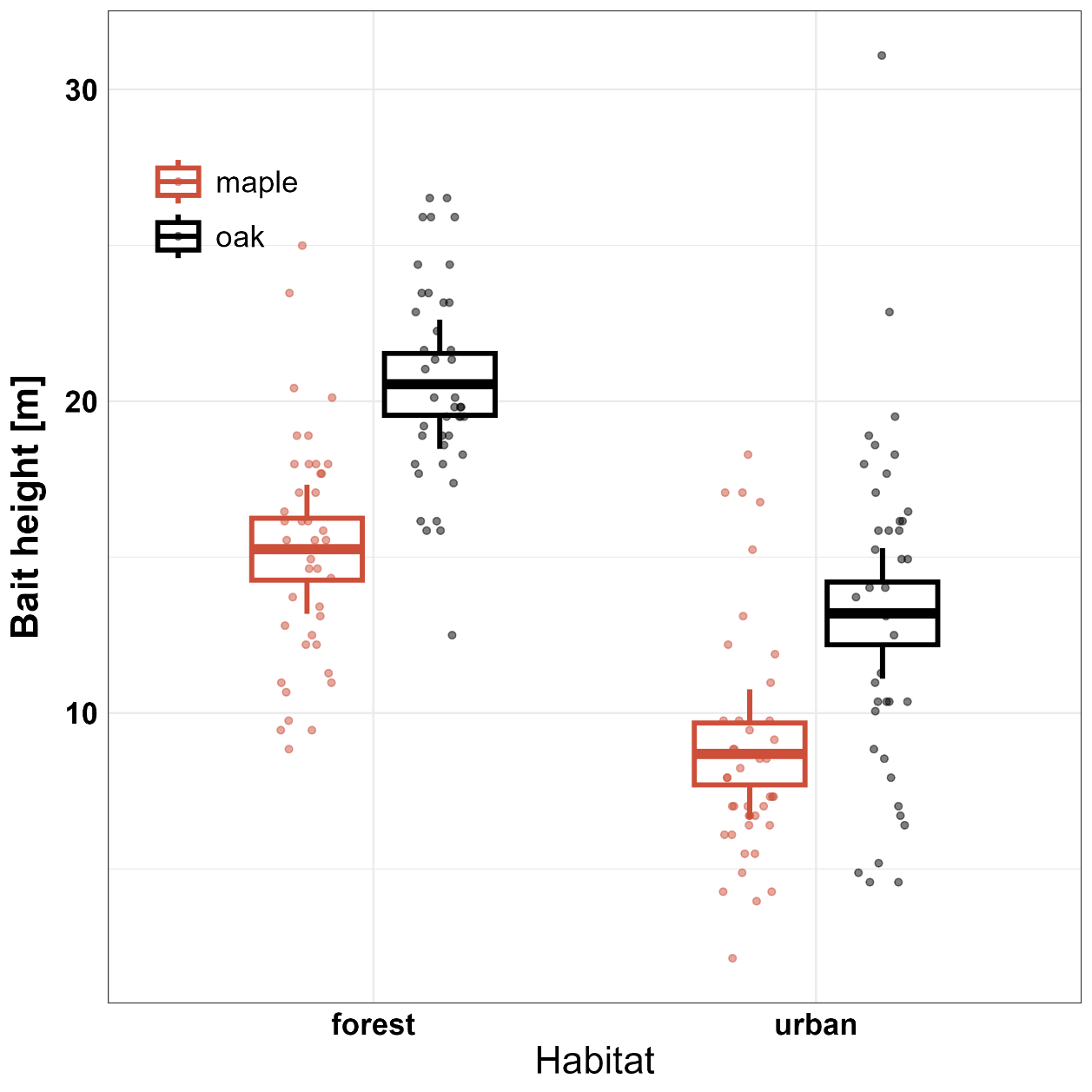


**Figure S1:** **Bait height varied with habitat and tree species**. Boxes are model-predicted means ± SE and whiskers are 95% confidence intervals. Points represent individual bait stations. See Text S3 for more information.

**
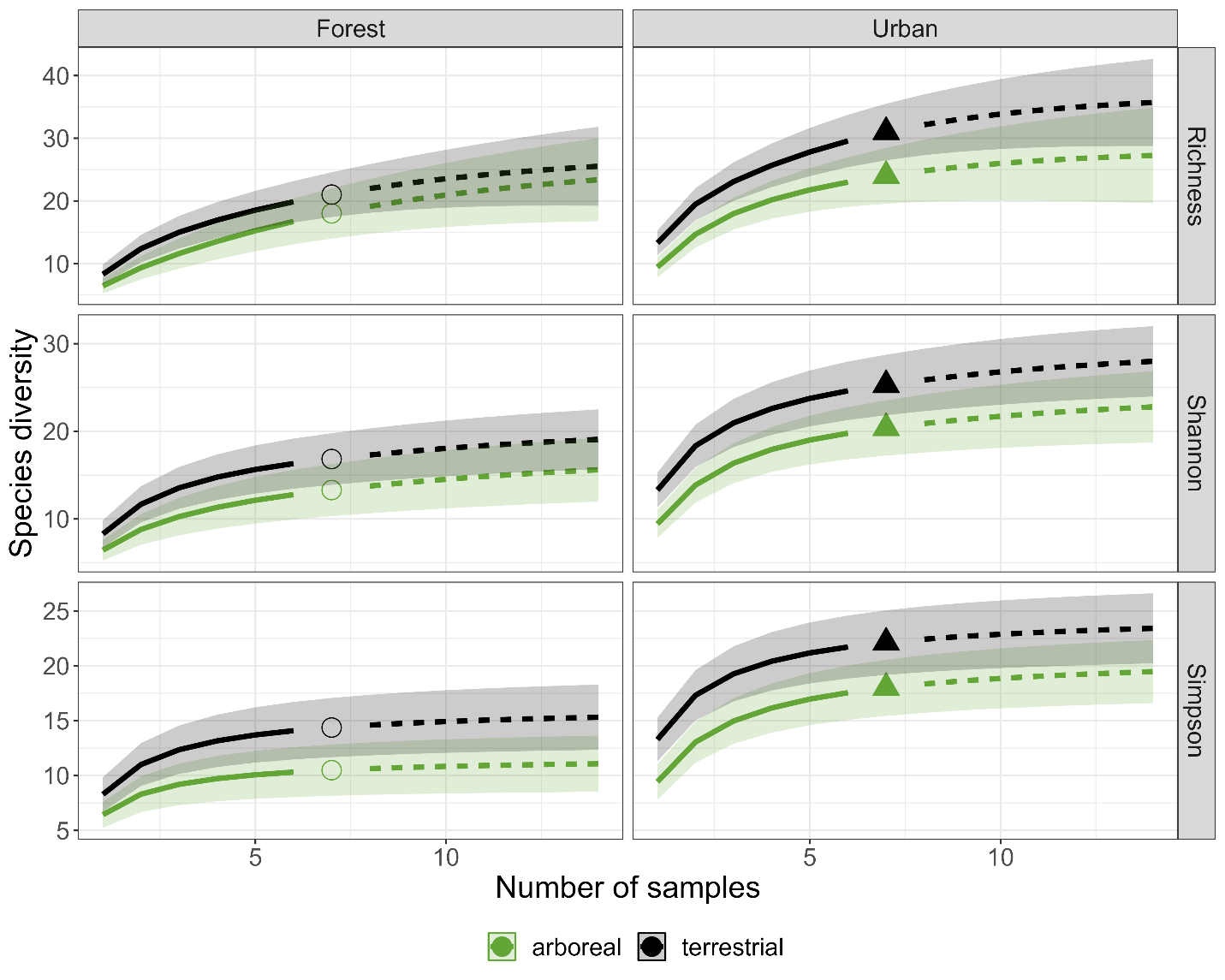
**

**Figure S2:** **Ant diversity at baits did not significantly differ between habitats and strata.** Sample-based rarefaction (solid lines) and extrapolation curves (dashed lines) of ant species richness (top), Shannon diversity (middle), and Simpson diversity (bottom) across vertical strata (canopy/ground) and habitat types (forest/urban). Shaded regions represent 95% confidence intervals. Observed species richness is denoted by a circle (forest) or triangle (urban).

**
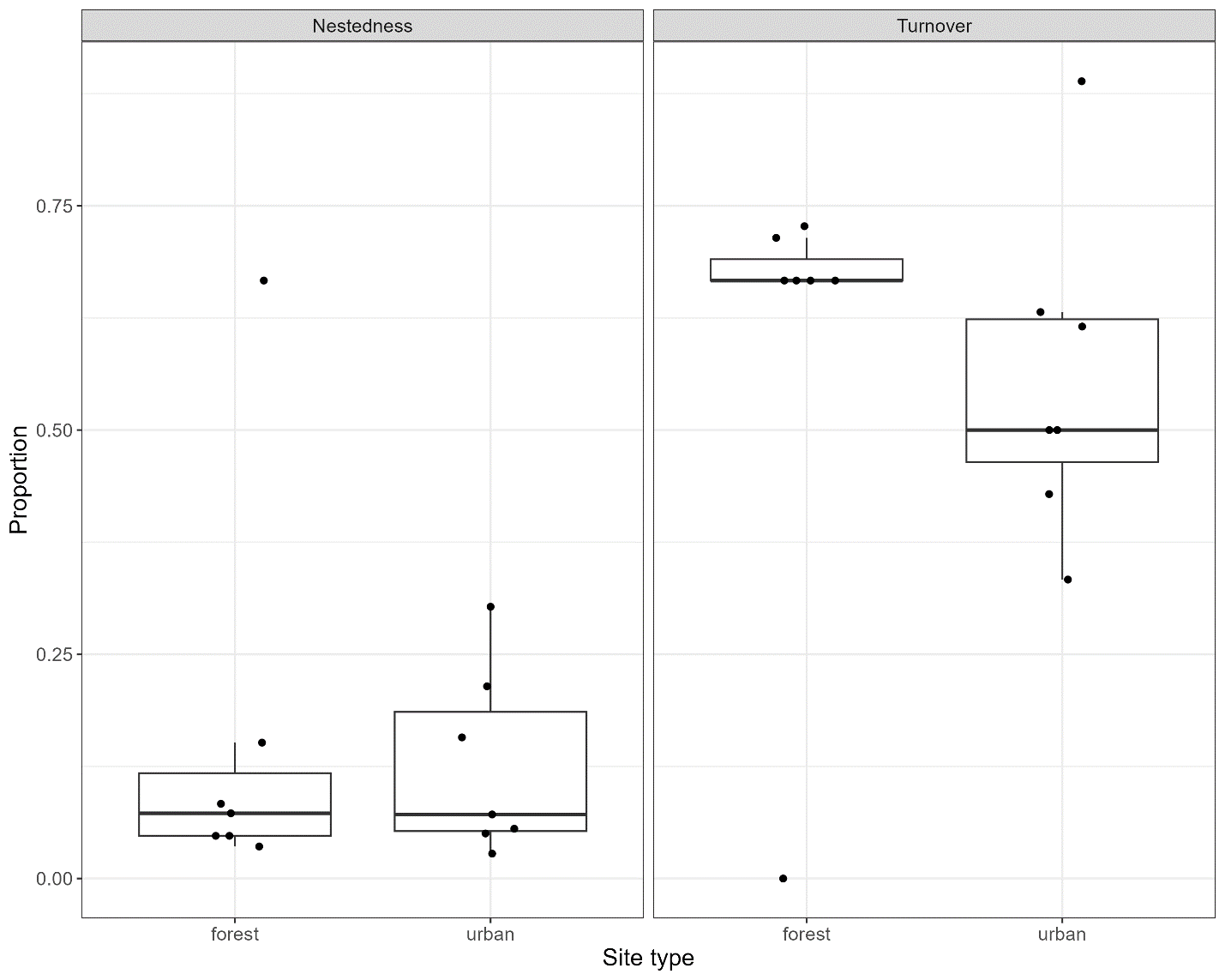
**

**Figure S3:** **Changes in ant communities between the ground and canopy were due to species turnover.** The contribution of nestedness and species turnover to community dissimilarity between vertical strata at forest and urban sites. Each point represents a site.


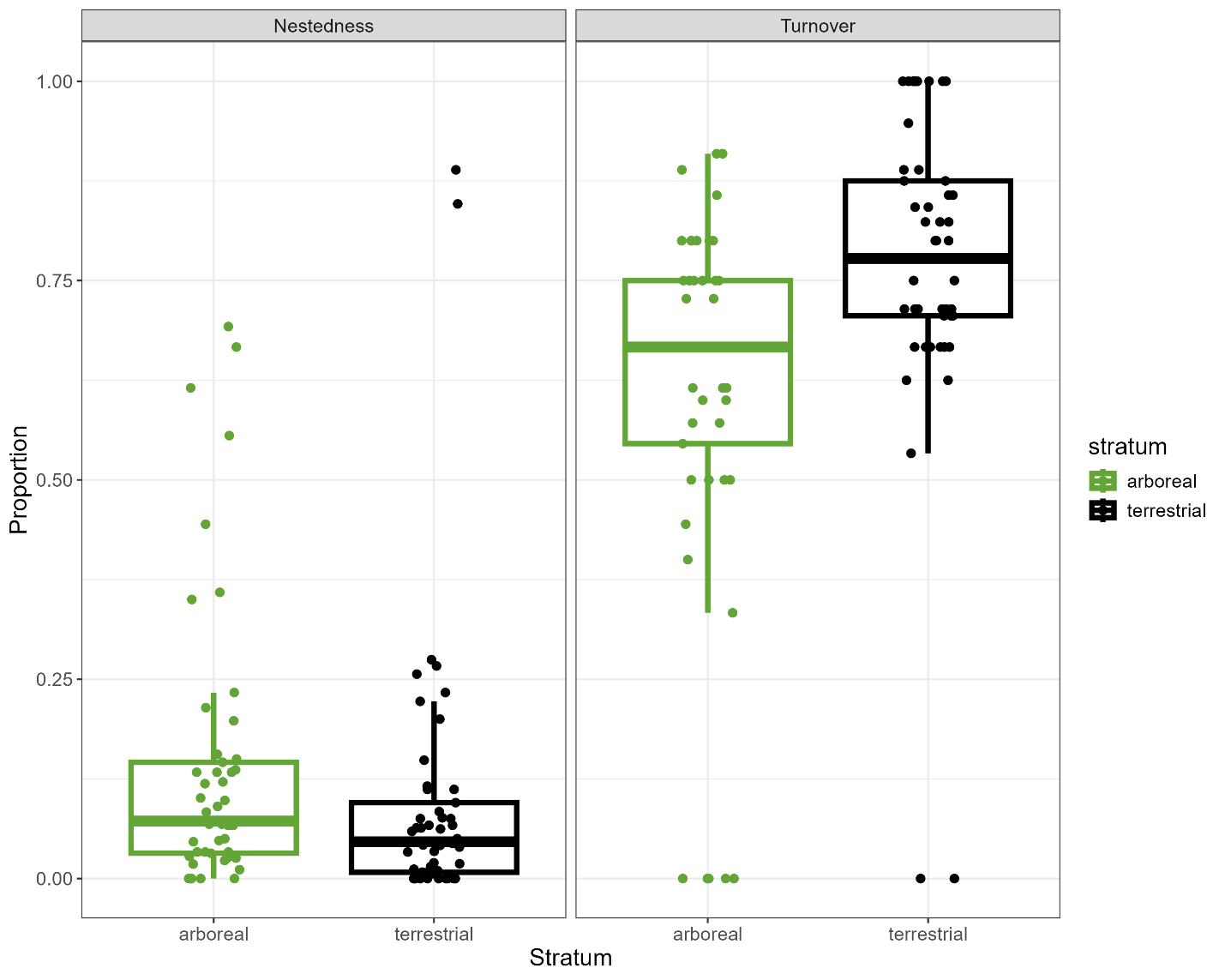


**Figure S4:** **Changes in ant communities between the forest and urban habitats were due to species turnover.** The contribution of nestedness and species turnover to community dissimilarity habitats at in the canopy and on the ground. Each point represents a comparison between an urban and a forest site.

**
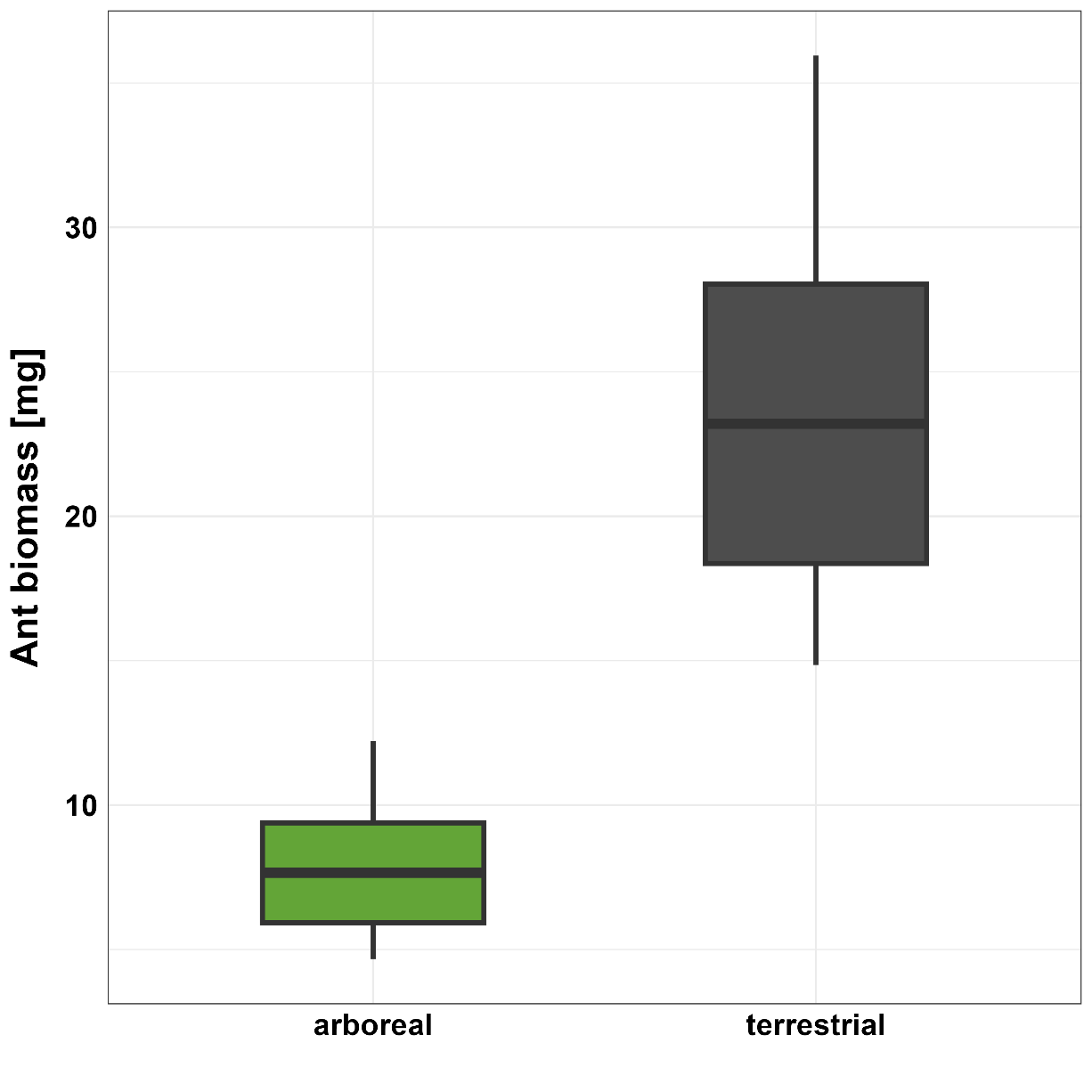
**

**Figure S5:** **Ant biomass was significantly greater on the ground than in the canopy.** Across habitat types, significantly more ant biomass was collected on the ground at each tree (ratio = 0.358, std. error = 0.054, df = 243, t-value = -6.84, p < 0.001). Boxes are model-predicted mean biomass per tree ± SE and whiskers are 95% confidence intervals.

**References**

AntWiki. 2014a. “Key to US Pseudomyrmex Species.” AntWiki. 2014. https://www.antwiki.org/wiki/Key_to_US_Pseudomyrmex_species.

———. 2014b. “Key to US Tapinoma Species.” AntWiki. 2014. https://antwiki.org/wiki/Key_to_US_Tapinoma_species.

———. 2017. “Key to US Tetramorium Species.” AntWiki. 2017. https://www.antwiki.org/wiki/Key_to_US_Tetramorium_species.

———. 2018. “Key to North American Lasius Species.” AntWiki. 2018. https://antwiki.org/wiki/Key_to_North_American_Lasius_Species.

———. 2019. “Key to US Monomorium Species.” AntWiki. 2019. https://antwiki.org/wiki/Key_to_US_Monomorium_species.

Creighton, W. S. 1950. “The Ants of North America.” *Bulletin of the Museum of Comparative Zoology* 104: 1–585.

DeMarco, Bernice B. 2015. “Phylogeny of North American Aphaenogaster Species (Hymenoptera:Formicidae) Reconstructed with Morphological and DNA Data.” Ph.D., East Lansing, MI: Michigan State University. https://antwiki.org/wiki/images/9/9f/Demarco%2C_B.B._2015._PhD_thesis.pdf.

Fisher, Brian L., and Stefan P. Cover. 2007. *Ants of North America: A Guide to the Genera*. Berkeley: University of California Press.

Francoeur, A. 1973. “Révision Taxonomique Des Espèces Néarctiques Du Groupe Fusca, Genre Formica (Formicidae, Hymenoptera).” *Mémoires de La Société Entomologique Du Québec* 3: 1–316.

Gregg, Robert E. 1958. “Key to the Species of Pheidole (Hymenoptera: Formicidae) in the United States.” *Journal of the New York Entomological Society* 66 (1/2): 7–48.

Hamm, Christopher A. 2010. “Multivariate Discrimination and Description of a New Species of Tapinoma from the Western United States.” *Annals of the Entomological Society of America* 103 (1): 20–29. https://doi.org/10.1603/008.103.0104.

Kallal, Robert J., and John S. LaPolla. 2012. “Monograph of Nylanderia (Hymenoptera: Formicidae) of the World, Part II: Nylanderia in the Nearctic.” *Zootaxa* 3508 (1): 1. https://doi.org/10.11646/zootaxa.3508.1.1.

MacGown, Joe A. 2022. “Ants (Formicidae) of the Southeastern United States - Keys to Subfamilies, Genera, and Species in or Possible in the Southeastern United States.” Missipippi Entomological Museum. 2022. https://mississippientomologicalmuseum.org.msstate.edu/Researchtaxapages/Formicidaepages/Identification.Keys.htm.

Ortiz-Sepulveda, Claudia M., Bert Van Bocxlaer, Andrés D. Meneses, and Fernando Fernández. 2019. “Molecular and Morphological Recognition of Species Boundaries in the Neglected Ant Genus Brachymyrmex (Hymenoptera: Formicidae): Toward a Taxonomic Revision.” *Organisms Diversity & Evolution* 19 (3): 447–542. https://doi.org/10.1007/s13127-019-00406-2.

Pacheco, Jose A, and William P Mackay. 2013. *The Systematics and Biology of the New World Thief Ants of the Genus Solenopsis (Hymenoptera: Formicidae)*. Lewiston, New York, USA: The Edwin Mellen Press.

Trager, James C, Joe A MacGown, and Matthew D Trager. 2007. “Revision of the Nearctic Endemic Formica Pallidefulva Group.” *Memoirs of the American Entomological Institute*, Advances in ant systematics (Hymenoptera: Formicidae): homage to E. O. Wilson – 50 years of contributions., 80: 610–36.

Ward, Philip S, and Bonnie B Blaimer. 2022. “Taxonomy in the Phylogenomic Era: Species Boundaries and Phylogenetic Relationships among North American Ants of the Crematogaster Scutellaris Group (Formicidae: Hymenoptera).” *Zoological Journal of the Linnean Society* 194 (3): 893–937. https://doi.org/10.1093/zoolinnean/zlab047.
