# Supplementary material for: Urbanization strengthens vertical stratification of ant nutrient preferences in a temperate forest ecosystem
Source: PLoS One. 2025 Mar 28;20(3):e0320380. doi: 10.1371/journal.pone.0320380 (PMC11952225; doi:10.1371/journal.pone.0320380)
Supplement: Attachment — Submitted filename: 20240416_Ants^L0Urbanization_MSPlosOne_Reviewed2.docx [file pone.0320380.s002.docx]

*PLOS One*: Research Article

**Urbanization strengthens vertical stratification of ant nutrient preferences in a temperate forest ecosystem**

Michelle Kirchner^1,2^, Lucie Ciccone^2*^, Clyde Sorenson^1^, Elsa Youngsteadt^2,3^

^1^Department of Entomology and Plant Pathology, North Carolina State University,100 Derieux Pl, Raleigh, NC, USA 27695. ^2^Department of Applied Ecology, North Carolina State University, 100 Eugene Brooks Ave, Raleigh, NC, USA 27695. ^3^Center for Geospatial Analytics, North Carolina State University, Raleigh, NC, USA 27695.

Corresponding author: Michelle Kirchner, email: [mlkirchn@ncsu.edu](mailto:mlkirchn@ncsu.edu)

Open Research Statement: Data and code are provided as private-for-peer review at the following link: <https://datadryad.org/stash/share/RMx3r2QT7CwzD-sc0jXxzM-Nhb-bd3qGkcZQh6F2JBU>

Key words: arboreal; canopy; carbohydrates; compensation hypothesis;; Formicidae; nutritional ecology; protein; urban ecology; macronutrients; food waste.

^*^Current affiliation: Student Conservation Association, 1310 N Courthouse Rd, Arlington VA, USA, 22201.

**Abstract**

Resource and nutrient availability vary spatially, consequently influencing animal foraging patterns. Under the compensation hypothesis, animals should preferentially forage for the most limiting nutrient in the environment. Animal nutrient preferences have been well studied in the tropics, where terrestrial and arboreal fauna are clearly differentiated and limited by different nutrients. In temperate forests, vertical stratification of the fauna may be less pronounced and its role in foraging ecology is poorly understood. Here, we examine nutrient preference patterns over a vertical gradient in temperate forests and nearby urban centers in North Carolina, USA. Using a bait-choice experiment and novel bait design, we measured ant community nutrient preferences in the canopy and on the ground of 83 trees across 14 sites and assessed ant diversity and community composition. Ant diversity did not differ across the vertical strata or habitat types, but species turnover altered community composition to create four distinct assemblages. In temperate forests, ants did not prefer a particular nutrient in either stratum, likely due to extensive foraging across strata. In urban habitats, however, ant nutrient preferences matched patterns well known from tropical systems: arboreal animals preferred protein, while terrestrial animals preferred carbohydrates. Rather than stratum-specific nutrient limitations, however, we attribute the differences in urban preference patterns to changes in native species’ foraging intensity and the addition of uniquely urban species with specific nutrient preferences. These results underscore the necessity of testing ecological hypotheses across biomes and suggest that urbanization may produce established ecological patterns via novel mechanisms.

**Introduction**

Animals forage to meet their nutritional needs to survive and reproduce. Because nutrient availability varies across landscapes and trophic levels, animals must adjust their feeding behaviors to regulate nutrient intake toward an optimum level (Raubenheimer, Simpson, and Mayntz 2009). According to the compensation hypothesis, animals preferentially forage for the most limiting nutrient in the environment (Kaspari et al. 2012). This “bottom-up” effect can influence foraging behavior at small and large spatial scales, with consequences for community composition, trophic webs, and species interactions (e.g., Yanoviak and Kaspari 2000; Lasmar et al. 2021; Law and Parr 2020; Davidson 2005; 1997).

In forests, nutrient availability varies along a vertical gradient due to fundamental differences in the dominant food webs across strata (Yanoviak and Kaspari 2000). Living vegetation in the forest canopy serves as the base of the arboreal “green food web”, where C:N ratios can exceed 40:1 (Foster and Bhatti 2017). In contrast, the forest floor is fed by detritus of the “brown food web”, and decomposition increases nitrogen levels, such that C:N ratios are 10:1 (Moore et al. 2004). Following the compensation hypothesis, arboreal animals should preferentially forage for protein-rich foods to offset the relatively low nitrogen availability, but because nitrogen is plentiful in the “brown food web,” animals in the leaf litter should prefer carbohydrate-rich foods (INSERT AT LEAST A REFERENCE HERE). Indeed, canopy-dwelling primates expend more relative foraging effort on high-nitrogen resources than expected based on their caloric value (Takahashi et al. 2019), and tropical ant communities prefer protein baits in the canopy and sugar baits on the ground (e.g., Kaspari et al. 2012; Law and Parr 2020; but see Lasmar et al. 2023). While vertical stratification of animal nutrient preferences is relatively well studied in tropical forests, we know little about patterns in temperate forests (but see Fowler, Lessard, and Sanders 2014). Even though the vertical gradient in relative carbon and nitrogen availability is similar in foliage and leaf litter across temperate broadleaf and tropical forests (McGroddy, Daufresne, and Hedin 2004), nutrient availability at higher trophic levels may depend on site-level factors, such as local climate, physiography, biotic communities, and disturbance (Foster and Bhatti 2017; Theis, Quévreux, and Loreau 2022). Given the climatic and structural differences between temperate and tropical forests that could result in distinct nutrient dynamics, it is unclear whether animal nutrient preference patterns known from tropical forests should persist in a temperate setting, leaving a key gap in our grasp of trophic ecology in temperate forests.

A clearer understanding of temperate forest ecology is critical at a time when temperate forests, already the most heavily fragmented forest biome globally (Haddad et al. 2015), are expected to suffer significant losses from urbanization in the near future (Chen et al. 2020). Urbanization impacts every level of the trophic web by altering ambient and external resources quality and availability (El-Sabaawi 2018). Although foliar and soil C:N ratios are similar between urban and rural forests (Wei and He 2021; Caron et al. 2023), animals in cities also have access to anthropogenic food inputs in the form of food waste and rubbish (Carpenter and Savage 2021). These additional resources tend to be heavily biased toward carbohydrates, altering nutrient availability such that urban animals shift their dietary preferences and nutrient intake (Carpenter and Savage 2021; Wist, Stolter, and Dausmann 2022; Penick, Savage, and Dunn 2015). The addition of carbohydrate-rich anthropogenic resources on the urban ground may balance the high nitrogen content of the terrestrial “brown food web,” but this possibility has not been tested in the context of the compensation hypothesis.

To address these knowledge gaps, we assessed ant community nutritional preferences on the ground and in the tree canopy of temperate deciduous forests and nearby urban areas. Ants (Hymenoptera: Formicidae) are an established model for nutritional ecology (e.g., Lasmar et al. 2023), as they are abundant in both epigaeic and arboreal habitats and exhibit striking diversity across trophic levels (Kaspari et al. 2012; Davidson 2005). We characterized ant diversity, community composition, and nutrient preference (carbohydrates and proteins) in both habitats (forest and urban) and vertical strata (canopy and ground). We expected community composition to follow established patterns, wherein temperate ants are more diverse on the ground than in the canopy (Floren, Wetzel, and Staab 2014), and non-urban sites are more diverse than urban sites (e.g., Santos, Delabie, and Queiroz 2019). The following nutritional predictions on ant ecology were tested here:

1. According to the compensation hypothesis, ants foraging in the temperate forest canopy will prefer protein, while those on the temperate forest ground will prefer carbohydrates.

2. Urbanization will eliminate nutrient preferences on the ground, but nutrient preferences in the canopy will remain unchanged.

**Methods**

*Study sites*

Field work took place in the Piedmont ecoregion of central North Carolina (NC), USA, which falls under a temperate-group Köppen-Geiger classification (Cfa: humid subtropical) (Beck et al. 2018) with mean monthly temperatures ranging from -2.8°C in January to 31.7°C in July (Griffith et al. 2002). The Piedmont ecoregion of the southeastern United States covers ~166,000km^2^ and contains major metropolitan areas, including Atlanta, Charlotte, and Raleigh-Durham. The region is under intense urbanization pressure, and developed land is expected to triple between 2009 and 2060 (Terando et al. 2014).

At seven forest and seven urban sites, we selected three healthy, mature trees each of two common species: white oak (*Quercus alba* L.) and red maple (*Acer rubrum* L.) (Fig. 1a). Forest sites were in protected, secondary mixed hardwood forests in state parks, game lands, or private property, and urban sites were in metropolitan areas of Raleigh and Durham on city-owned rights-of-way, which included street medians, residential lawns, parking lots, commercially maintained landscaping, and sidewalks. Sites were separated by at least 2km, and trees within a site were at least 20m apart. All sampling was done with permits from NC State Parks, NC Wildlife Resource Commission, City of Raleigh, City of Durham, or owner permission.

*Ant sampling and biomass*

Ants were sampled, one dry day per site, between June 30 and August 9, 2021, using a novel bait station design (Fig. 1b; see Text S1 for details). Each bait station held a pair of baits, and each bait contained ~6cm^3^ of either a carbohydrate-rich (strawberry preserves, Smucker’s^®^) or protein-rich food (tuna, StarKist^®^ chunk light in water, drained) (Table S1). At each tree, we placed four bait stations, two in the canopy (mean height: 14.6m, range: 2.1–31.1m) and two on the ground (5m N and S of the base of the tree) (Fig. 1c) between 9:00 and 11:30am. After 4 and 8hr, we collected all ants on each bait, by putting them into vials with 70% ethanol. Ants were identified to species using genus-specific keys (e.g., Ortiz-Sepulveda et al. 2019; Ward and Blaimer 2022; Appendix S1: Table S2). Vouchers were deposited in the NC State University Insect Museum. To analyze nutrient preference by ant species, we sorted all ants from each bait by species, dried the specimens (24h in a 55°C dry block followed by 48h in a 55°C drying oven) and weighed them to the nearest 0.01mg with an analytical balance (Mettler Toledo MS105DU, readable to 0.01mg) or microbalance (Mettler Toledo XP6, readable to 1µg).

*Data analysis*

Data analyses were performed in *R* 4.2.2 and *RStudio* 2023.3.1.446 (R Core Team 2022; Posit team 2023).

*Diversity and community composition* – We pooled all baits within each combination of site and stratum (N=28) and recorded each ant species’ presence or absence. To assess differences in ant species diversity between strata (arboreal/terrestrial) and site types (forest/urban), we performed a sample-based rarefaction and extrapolation using *iNEXT* 3.0.0 (Hsieh, Ma, and Chao 2016; Chao et al. 2014). Then, we removed singleton species and tested for changes in community composition in *vegan* 2.6-4 (Oksanen et al. 2022). First, we conducted permutational multivariance analysis of variance (PERMANOVA) using Jaccard dissimilarity indices. We included the effects of stratum, site type, and their interaction, and an additive effect of site as predictors in “adonis2”, performed 999 permutations for each test, and assessed the marginal effects of each term. We calculated pairwise comparisons between each level of the stratum*site type interaction by iteratively passing the appropriate subset of the distance matrix to “adonis2”, performing 10,000 permutations of each test, and then, adjusted for multiple comparisons using Benjamini & Hochberg’s (1995) method for controlling the false discovery rate (public communications, Maurice Codespoti Goodman, pairwise_permanova.R, accessed June 6, 2023, https://gist.github.com/mcgoodman/58c9d1257fd1625954a4ffa1c3301939). We also tested for multivariate homogeneity of dispersion around each group’s median (PERMDISP2) with “betadisper”. To visualize shifts in community composition, we performed non-metric multidimensional scaling (NMDS) on the species occurrence Jaccard distance matrix, ordinated on three dimensions using “ordiplot3d” in *vegan3d* 1.1-2 (Oksanen et al. 2023). Finally, to clarify the role of species replacement versus loss in separating the canopy and ground communities, we computed species turnover and nestedness between the canopy and ground for each site using “beta.pair” in *betapart* 1.6 (Baselga et al. 2023).

*Nutritional preference* – We recorded total ant biomass in each bait and pooled baits such that each of the 83 trees was represented by four biomass data points: arboreal protein, arboreal carbohydrate, terrestrial protein, and terrestrial carbohydrate. To account for ants removed for identification, we calculated the average weight of a single individual of each species and used it to add the estimated biomass of each of the 1,292 pinned individuals back into their sample of origin. For three species for which we could not calculate an average weight, we used the calculated average weight of a congener of similar size (Appendix S1: Table S3), and we excluded one species, *Pseudomyrmex ejectus* (Smith), which was represented by a single individual across all baits. To stabilize variance, we log+1 transformed the total biomass estimates. We then used a linear mixed model constructed with *lme4* 1.1-31 (Bates et al. 2015) to assess how ant nutrient preference shifted between site types and strata. We modeled the transformed biomass as a function of stratum, site type, nutrient, and their interactions with random intercepts of site and tree. We checked model fit with diagnostic plots and tests in *DHARMa* 0.4.6 (Hartig and Lohse 2022) and tested significance using Type II Wald χ^2^ tests in *car* 3.1-1 (Fox and Weisberg 2019). We calculated planned simple main effect contrasts for the three-way interaction between stratum, site type, and nutrient with *emmeans* 1.8.4-1 (Lenth et al. 2023). We report back-transformed marginal means and their errors. We accounted for multiple comparisons using multivariate *t* probabilities (Lenth et al. 2023). To visualize community nutrient preference patterns, we calculated every species’ biomass for each combination of site type, stratum, and nutrient and plotted one bipartite network per site type using *bipartite* 2.18 (Dormann et al. 2009; Dormann, Gruber, and Fründ 2008).

Finally, we tested if ant community-level differences in nutrient use between forest and urban sites could arise from the following non-exclusive scenarios: (a) consistent changes in the behavior of many individual species that occur in both site types, (b) shifts in the behavior of one or a few species that dominate the community biomass, or (c) the distinct preferences of species that were predominantly or uniquely urban. To understand how individual species’ preferences shifted with urbanization (scenario (a)), we calculated the proportion of each species’ biomass collected from arboreal protein and terrestrial carbohydrate baits within each site. We only included species which occurred in both forest and urban sites (N=16) and fit a beta regression model constructed with *glmmTMB* 1.1.5 (Brooks et al. 2017). We modeled the proportions as a function of site type with a random intercept of species, checked model fit with diagnostic plots, and tested statistical significance using Type II Wald χ^2^ tests in *car* 3.1-1. For scenario (b), we considered species to be dominant if they represented more than 10% of the total biomass at a given site type. For each of the urban dominant species, we visually assessed the two bipartite networks for evidence of behavioral changes between strata and site types. For scenario (c), we considered two assemblages: uniquely urban species and tropical species. We repeated the log+1 transformed linear mixed model with fixed effects of stratum, nutrient, and their interaction and random intercepts of site and tree to understand how the addition of these species to the urban matrix could contribute to the observed preference patterns.

**Results**

After excluding missing and damaged baits, we retrieved 1321 individual baits (out of 1344) from 83 trees (42 forest, 41 urban). Ants occupied 786 baits across all sites and trees and were detectable in both strata (arboreal: 300 baits, terrestrial: 486). We collected a total of 47 ant species in 17 genera and 5 subfamilies (Appendix S1: Table S4), representing more than a quarter of the 164 species known from this region (Guénard et al. 2012).

*Diversity and community composition*

We observed the most species in the urban terrestrial habitat (31 spp.) and the fewest in the forest arboreal habitat (18), and all habitats had a similar number of unique, non-singleton species (two species each in urban arboreal, forest arboreal and forest terrestrial; three species in urban terrestrial). In urban sites, 14% of species were detected only at arboreal baits, 36% terrestrial, and 50% in both strata. In forests, 19% of species were arboreal, 31% terrestrial, and 50% were recorded in both habitats. Chao2 asymptotic estimators of species richness were similar across the habitats (urban arboreal: 28.2 spp., urban terrestrial: 37.1, forest arboreal: 26.7, forest terrestrial: 27.9) and shared overlapping 95% confidence intervals (Fig. 2a). Other measures of diversity followed the same pattern (Appendix S1: Fig S1). Based on these results, and reasonably high sample coverage in all four habitats (urban arboreal: 0.914, urban terrestrial: 0.913, forest arboreal: 0.826, forest terrestrial: 0.882), we conclude that ant diversity at baits did not differ between strata and site types.

Ant community composition depended on the site type*stratum interaction (PERMANOVA: F_(1,12)_=2.62, R^2^=0.047, p=0.013) and the additive effect of site (PERMANOVA: F_(12,12)_=1.81, R^2^=0.388, p=0.001). Because the community variances did not differ (PERMDISP2: F_(3,24)_=0.546, p=0.655), we attribute the significant PERMANOVA to differences in the community centroids. All pairwise comparisons between levels of the site type*stratum interaction were statistically significant (Fig. 2b, Appendix S1: Table S5). In both forests and urban areas, community differences between the canopy and ground were largely due to species turnover rather than nestedness (forest: average nestedness=0.16, average turnover=0.59; urban: average nestedness=0.13, average turnover=0.56) (Appendix S1: Fig S2).

*Nutrient preference*

As expected for a temperate system, we collected significantly more ant biomass on the ground (Table 1; Fig S3), but 33.6% of our total ant biomass was found in the canopy, suggesting that the tree canopy is still a valuable resource for temperate ant communities. Species that foraged both in the canopy and on the ground accounted for 98% of biomass in forest and urban sites. Community-wide nutrient preferences differed among strata and site types (Table 1). In forests, ants showed no nutrient preference in either stratum. In urban sites, however, ants in the canopy preferred protein (p<0.001), while ants on the ground tended to prefer carbohydrates (p=0.082) (Fig. 3; Table S6). We investigated three scenarios by which these distinct, community-wide preference patterns could occur.

*Scenario (a): Consistent shifts in multiple species’ behaviors.* At the species level, nutrient preference patterns were consistent across habitat types within each stratum (terrestrial: χ^2^=0.003, df=1, p=0.958; arboreal: χ^2^=1.73, df=1, p=0.188), indicating that the urban preference shift was not due to a consistent, multi-species shift in nutrient use between forest and urban sites.

*Scenario (b): Shifts in dominant species’ behaviors.* The urban ant-nutrient bipartite network was more complex (connectance=0.639) than the forest network (connectance=0.577), because resources were less monopolized by single species. Nevertheless, we identified four dominant urban species (>10% of total urban biomass each). Of these, *Crematogaster ashmeadi* Mayr is responsible for the urban arboreal protein preference. *Crematogaster ashmeadi* foraged almost exclusively for arboreal protein in both site types but was absolutely and relatively more abundant in urban sites. We collected this species from a similar number of trees in each habitat type (20 forest, 22 urban), but its biomass measured over 5.5 times more in urban trees (forest: 243.5mg, urban: 1345.2mg), leading to more overall ant biomass in urban arboreal protein than urban arboreal carbohydrates. The other three biomass-dominant taxa did not appear to contribute to the forest-urban preference shift. *Formica subsericea* Say preferred terrestrial carbohydrates in both site types, and *Camponotus pennsylvanicus* (DeGeer) showed a slight protein preference in both strata and site types, but both taxa made up a similar or smaller share of community biomass in urban sites than forest sites. *Solenopsis invicta* Buren was much more abundant in the city (forest biomass: 0.6mg, urban biomass: 838.5mg), but foraged equally on carbohydrates and protein in both strata.

*Scenario (c): Addition of uniquely urban and tropical species.* The 17 species unique to urban sites significantly preferred terrestrial carbohydrates to terrestrial protein (stratum*nutrient: χ^2^=5.50, df=1, p=0.019; terrestrial C—terrestrial P: t_(72.6)_=3.76, p=0.0007), suggesting that the observed urban terrestrial preference for carbohydrates resulted from the addition of these species. Results were qualitatively similar when we considered only the 13 urban species that were native. Moreover, the three tropical species (*Brachymyrmex patagonicus* Mayr, *Linepithema humile* (Mayr), and *Solenopsis invicta*) showed no nutrient preference regardless of stratum (stratum*nutrient: χ^2^=0.282, df=1, p=0.596; nutrient: χ^2^=1.64, df=1, p=0.201). Thus, our data suggest that the addition of native species to the urban habitat is the main driver of the terrestrial carbohydrate preference (Fig. 4, see *Formica biophilica* Trager, *Lasius neoniger* Emery, and *Tapinoma* cf. *sessile* (Text S2)).

**Discussion**

We assessed community patterns of ant diversity and compared nutrient preferences across two vertical strata in temperate forests and an urban metropolis. Whereas tropical studies find strong vertical stratification of ant diversity and nutrient preference (PLEASE, INSERT REFERENCES FOR THESE STUDIES), we detected neither patterns in our temperate forest sites. In nearby urban trees, however, we did observe the vertically stratified nutrient preferences, described for many tropical systems, such that ants in the canopy preferred protein, while ants on the ground preferred carbohydrates (e.g., Kaspari et al. 2012; Law and Parr 2020) (Fig. 3).

Contrary to our predictions, ant diversity did not differ between vertical strata or site types. Each community (urban arboreal, urban terrestrial, forest arboreal, forest terrestrial) had a distinct species composition (Fig. 2, Appendix S1: Table S5) driven mainly by species turnover (Appendix S1: Fig S2). Surprisingly few studies make direct comparisons between urban and non-urban ant communities; our results are consistent with the few prior terrestrial studies that find no difference in alpha diversity between urban and non-urban sites (PLEASE, INSERT SOME REFERENCES), or peak diversity in suburban areas (Menke et al. 2011). However, our diversity results should be interpreted with caution because baiting does not sample the entire ant community (Antoniazzi et al. 2020).

The vertical stratification of C:N ratios at the producer level is similar in temperate and tropical forests (McGroddy, Daufresne, and Hedin 2004), but while tropical ants often expend more foraging effort on the limiting nutrient in each stratum, the temperate forest ant communities studied here contributed equal biomass to protein- and carbohydrate-rich baits, regardless of vertical stratum (Fig. 3). Similarly, Fowler, Lessard, and Sanders (2014) demonstrated a lack of vertical stratification in nutrient preferences across three habitat strata (ground, shrubs, and tree trunks) for the ants of Great Smoky Mountains National Park, less than 500km west of our study sites. However, due to regional or methodological differences, those communities distinctly preferred carbohydrates in every stratum.

The complete lack of nutrient preferences in our temperate forest ant communities likely reflects the ability of temperate ant species to forage flexibly in both strata. Whereas studies of tropical ant nutrient preferences found only 0 – 16% of species foraging in both strata (Yanoviak and Kaspari 2000; Law and Parr 2020; Lasmar et al. 2023), we detected 50% of forest species in both strata, and these species represented 98% of forest ant biomass. Even though canopy and ground communities were statistically distinct and harbored unique species, extensive cross-stratum foraging likely masked any influence of stratum-specific nutrient profiles at the community level. Stratum specialists may still be exposed to these limitations, but they represented only a small proportion of total forest ant biomass. Our results underscore the importance of testing ecological hypotheses in different contexts. Here, we show that a well-studied, relatively consistent trophic phenomenon in tropical forests does not translate in a temperate system.

However, in nearby urban trees, we recovered the vertically stratified nutrient preferences typical of tropical studies: ants in the canopy preferred protein, while those on the ground trended toward carbohydrates (Fig. 3). Urban ants thus matched the predictions of the compensation hypothesis and contradicted our prediction that anthropogenic carbohydrate subsidies (Carpenter and Savage 2021; Wist, Stolter, and Dausmann 2022) would eliminate this pattern in terrestrial urban ants. It is not surprising that nutrient preferences would shift in an urban matrix. Anthropogenic food inputs, patchy resource availability, and variable food quality can affect the trophic structure, niche breadth, dietary preference, and nutrient intake of a variety of urban animals (Penick, Savage, and Dunn 2015; Liang et al. 2020; Youngsteadt et al. 2015; Wist, Stolter, and Dausmann 2022). Still, we find it interesting that the community preferences in a novel urban habitat shifted to mimic patterns known from old-growth tropical forests. Nevertheless, it seems unlikely that the observed distribution of ants directly reflects stratum-specific nutrient limitations, because the urban ant community foraged across strata just as much as the forest community did. Our exploratory analyses instead suggest that urban ants’ preference for protein in the canopy was best explained by the increased foraging intensity of a native arboreal species, *Crematogaster ashmeadi,* in the urban habitat. This species always foraged on arboreal protein but was about 5.5 times more abundant on urban baits than on forest baits. Meanwhile, urban ants’ preference for carbohydrates on the ground was best explained by the presence of native species with strong carbohydrate preferences that were absent from the forest. Species that were found in both urban and forest sites did not change their preferences between habitats.

Thus, we cannot conclude, based on the distribution of ants as a whole, that nutrient availability is more steeply stratified in urban than forest sites. Instead, patterns of ant distribution consistent with the compensation hypothesis may have arisen incidentally from some combination of altered urban community composition, competition, microhabitat selection, and nutrient preferences of species within that community. For example, on the urban ground, anthropogenic carbohydrates subsidies might favor species already adapted to forage at low trophic levels, such as *F. biophilica* and *T.* cf. *sessile*, without exacerbating protein limitation (consistent with the economics hypothesis, Kaspari et al. 2012). In the canopy, high densities of honeydew-producing insects on stressed urban hardwoods (Smith, Dearborn, and Hutyra 2019; Frank and Backe 2023) might support larger colonies of *Cr. ashmeadi* that still have similar stoichiometry to those in the forest. We also acknowledge that by using complex foods as baits, other components such as water, fat, and micronutrients could have influenced ant behavior. Overall, we detected striking differences in ant biomass distribution between nutrients in urban and forest sites, but future studies adopting different approaches — such as stable isotope analyses and longer-term nutrient manipulations — are needed to parse the underlying mechanisms.

**Conclusion**

We provide evidence that temperate forest ant communities do not adhere to nutrient preference patterns predicted by the compensation hypothesis of nutrient availability. Instead, the majority of temperate ants commute freely between the canopy and ground, linking green and brown food webs in ways that tropical ants do not. These findings highlight the risk of making assumptions about ecosystem- and community-level processes across biomes. They also emphasize the need for more temperate forest ecology research to consider the tree canopy, where we detected more than one third of foraging ant biomass. We also show, for the first time, that urbanization in a temperate zone can alter ant community nutrient preferences to mirror tropical patterns, though likely due to different mechanisms. As more of our world is becoming urbanized, we are shifting community and ecosystem ecology in novel and unexpected ways. Future work that integrates nutritional ecology, trophic relationships, and nutrient availability in urban systems should provide valuable insights into some of the far-reaching effects of an urban landscape.

**Acknowledgments**

Kirsten Keleher and Kayla Beckwith assisted with sample processing, Peter Willadsen put his Big Shot™ skills to the test, James Trager was consulted on *Formica* and *Tapinoma* identifications, and Keary Dosier generously lent his drill press. Thank you to the Brandt family for private land access and genuine hospitality. Ed Corey (NC State Parks), Kim Radewicz (NC State Parks), Zac Cowan (NC State Parks), Scott Letchworth (NC State Parks), Chris Baranski (NC Wildlife Resources Commission), Sally Thigpen (Raleigh Parks, Recreation, and Cultural Resources), and Alex Johnson (Durham Urban Forestry Division) facilitated research permissions and site access. We appreciate the residents of Raleigh and Durham who tolerated this work in their front yards.

**Author Contributions**

MK, CS, and EY designed the study. MK and LC collected data. MK conducted all analyses and wrote the first draft of the manuscript. All authors provided critical review of drafts and gave final approval for publication.

**Conflict of Interest Statement**

The authors have no conflict of interest to declare.

**References**

Antoniazzi, Reuber, Diana Ahuatzin, Jaime Pelayo-Martínez, Liliana Ortiz-Lozada, Maurice Leponce, and Wesley Dáttilo. 2020. “On the Effectiveness of Hand Collection to Complement Baits When Studying Ant Vertical Stratification in Tropical Rainforests.” *Sociobiology* 67 (2): 213–22. https://doi.org/10.13102/sociobiology.v67i2.4909.

Baselga, Andres, David Orme, Sebastien Villeger, Julien De Bortoli, Fabien Leprieur, Maxime Logez, Sara Martinez-Santalla, et al. 2023. “Betapart: Partitioning Beta Diversity into Turnover and Nestedness Components.” R Package. R. https://cran.r-project.org/web/packages/betapart/index.html.

Bates, Douglas, Martin Mächler, Ben Bolker, and Steve Walker. 2015. “Fitting Linear Mixed-Effects Models Using Lme4.” *Journal of Statistical Software* 67 (October): 1–48. https://doi.org/10.18637/jss.v067.i01.

Beck, Hylke E., Niklaus E. Zimmermann, Tim R. McVicar, Noemi Vergopolan, Alexis Berg, and Eric F. Wood. 2018. “Present and Future Köppen-Geiger Climate Classification Maps at 1-Km Resolution.” *Scientific Data* 5 (1): 180214. https://doi.org/10.1038/sdata.2018.214.

Benjamini, Yoav, and Yosef Hochberg. 1995. “Controlling the False Discovery Rate: A Practical and Powerful Approach to Multiple Testing.” *Journal of the Royal Statistical Society: Series B (Methodological)* 57 (1): 289–300. https://doi.org/10.1111/j.2517-6161.1995.tb02031.x.

Brooks, Mollie, E., Kasper Kristensen, Koen Benthem J.,van, Arni Magnusson, Casper Berg W., Anders Nielsen, Hans Skaug J., Martin Mächler, and Benjamin Bolker M. 2017. “glmmTMB Balances Speed and Flexibility Among Packages for Zero-Inflated Generalized Linear Mixed Modeling.” *The R Journal* 9 (2): 378. https://doi.org/10.32614/RJ-2017-066.

Caron, Stephen, Sarah M. Garvey, Jonathan Gewirtzman, Kyle Schultz, Jennifer M. Bhatnagar, Charles Driscoll, Lucy R. Hutyra, and Pamela H. Templer. 2023. “Urbanization and Fragmentation Have Opposing Effects on Soil Nitrogen Availability in Temperate Forest Ecosystems.” *Global Change Biology* 29 (8): 2156–71. https://doi.org/10.1111/gcb.16611.

Carpenter, Melissa, and Amy M Savage. 2021. “Nutrient Availability in Urban Food Waste: Carbohydrate Bias in the Philadelphia–Camden Urban Matrix.” *Journal of Urban Ecology* 7 (1): juab012. https://doi.org/10.1093/jue/juab012.

Chao, Anne, Nicholas J. Gotelli, T. C. Hsieh, Elizabeth L. Sander, K. H. Ma, Robert K. Colwell, and Aaron M. Ellison. 2014. “Rarefaction and Extrapolation with Hill Numbers: A Framework for Sampling and Estimation in Species Diversity Studies.” *Ecological Monographs* 84 (1): 45–67. https://doi.org/10.1890/13-0133.1.

Chen, Guangzhao, Xia Li, Xiaoping Liu, Yimin Chen, Xun Liang, Jiye Leng, Xiaocong Xu, et al. 2020. “Global Projections of Future Urban Land Expansion under Shared Socioeconomic Pathways.” *Nature Communications* 11 (1): 537. https://doi.org/10.1038/s41467-020-14386-x.

Davidson, Diane W. 1997. “The Role of Resource Imbalances in the Evolutionary Ecology of Tropical Arboreal Ants.” *Biological Journal of the Linnean Society* 61 (2): 153–81. https://doi.org/10.1111/j.1095-8312.1997.tb01785.x.

———. 2005. “Ecological Stoichiometry of Ants in a New World Rain Forest.” *Oecologia* 142 (2): 221–31. https://doi.org/10.1007/s00442-004-1722-0.

Dormann, Carsten F., Jochen Fründ, Nico Blüthgen, and Bernd Gruber. 2009. “Indices, Graphs and Null Models: Analyzing Bipartite Ecological Networks.” *The Open Ecology Journal* 2 (1). https://benthamopen.com/ABSTRACT/TOECOLJ-2-1-7.

Dormann, Carsten F., Bernd Gruber, and Jochen Fründ. 2008. “Introducing the Bipartite Package: Analysing Ecological Networks.” *R News* 8 (2): 8–11.

El-Sabaawi, Rana. 2018. “Trophic Structure in a Rapidly Urbanizing Planet.” *Functional Ecology* 32 (7): 1718–28. https://doi.org/10.1111/1365-2435.13114.

Floren, Andreas, Waltraud Wetzel, and Michael Staab. 2014. “The Contribution of Canopy Species to Overall Ant Diversity (Hymenoptera: Formicidae) in Temperate and Tropical Ecosystems.” *Myrmecological News* 19: 65–74.

Foster, Neil W., and Jagtar S. Bhatti. 2017. “Forest Soils: Nutrient Cycling.” In *Encyclopedia of Soil Science*, edited by Rattan Lal, 3rd ed., 2:966–68. Boca Raton, FL, USA: Taylor & Francis Group.

Fowler, David, Jean-Philippe Lessard, and Nathan J. Sanders. 2014. “Niche Filtering Rather than Partitioning Shapes the Structure of Temperate Forest Ant Communities.” *Journal of Animal Ecology* 83 (4): 943–52. https://doi.org/10.1111/1365-2656.12188.

Fox, John, and Sanford Weisberg. 2019. *An R Companion to Applied Regression*. 3rd ed. Thousand Oaks, CA, USA: Sage Publications. https://socialsciences.mcmaster.ca/jfox/Books/Companion/.

Frank, Steven D., and Kristi M. Backe. 2023. “Effects of Urban Heat Islands on Temperate Forest Trees and Arthropods.” *Current Forestry Reports* 9 (1): 48–57. https://doi.org/10.1007/s40725-022-00178-7.

Griffith, Glenn E., James M. Omernik, Jeffrey A. Comstock, Michael P. Schafale, W. Henry McNab, David R. Lenat, Trish F. MacPherson, James B. Glover, and Victor B. Shelburne. 2002. “Ecoregions of North Carolina and South Carolina.” Data and Tools. Reston, Virginia: U.S. Geological Survey. https://www.epa.gov/eco-research/ecoregion-download-files-state-region-4.

Guénard, Benoit, Katherine A. Mccaffrey, Andrea Lucky, and Rob R. Dunn. 2012. “Ants of North Carolina: An Updated List (Hymenoptera: Formicidae).” *Zootaxa* 3552 (1): 1–36. https://doi.org/10.11646/zootaxa.3552.1.1.

Haddad, Nick M., Lars A. Brudvig, Jean Clobert, Kendi F. Davies, Andrew Gonzalez, Robert D. Holt, Thomas E. Lovejoy, et al. 2015. “Habitat Fragmentation and Its Lasting Impact on Earth’s Ecosystems.” *Science Advances* 1 (2): e1500052. https://doi.org/10.1126/sciadv.1500052.

Hartig, Florian, and Lukas Lohse. 2022. “DHARMa: Residual Diagnostics for Hierarchical (Multi-Level / Mixed) Regression Models.” R Package. R. https://cran.r-project.org/web/packages/DHARMa/index.html.

Hsieh, T. C., K. H. Ma, and Anne Chao. 2016. “iNEXT: An R Package for Rarefaction and Extrapolation of Species Diversity (Hill Numbers).” *Methods in Ecology and Evolution* 7 (12): 1451–56. https://doi.org/10.1111/2041-210X.12613.

Kaspari, Michael, David Donoso, Jane A. Lucas, Taylor Zumbusch, and Adam D. Kay. 2012. “Using Nutritional Ecology to Predict Community Structure: A Field Test in Neotropical Ants.” *Ecosphere* 3 (11): art93. https://doi.org/10.1890/ES12-00136.1.

Lasmar, Chaim J., Tom R. Bishop, Catherine L. Parr, Antônio C. M. Queiroz, Fernando A. Schmidt, and Carla R. Ribas. 2021. “Geographical Variation in Ant Foraging Activity and Resource Use Is Driven by Climate and Net Primary Productivity.” *Journal of Biogeography* 48 (6): 1448–59. https://doi.org/10.1111/jbi.14089.

Lasmar, Chaim J., Tom R. Bishop, Catherine L. Parr, Antônio C. M. Queiroz, Icaro Wilker, Rodrigo M. Feitosa, Fernando A. Schmidt, and Carla R. Ribas. 2023. “Testing the Context Dependence of Ant Nutrient Preference across Habitat Strata and Trophic Levels in Neotropical Biomes.” *Ecology*, e3975. https://doi.org/10.1002/ecy.3975.

Law, Stephanie J., and Catherine Parr. 2020. “Numerically Dominant Species Drive Patterns in Resource Use along a Vertical Gradient in Tropical Ant Assemblages.” *Biotropica* 52 (1): 101–12. https://doi.org/10.1111/btp.12743.

Lenth, Russell V., Ben Bolker, Paul Buerkner, Iago Giné-Vázquez, Maxime Herve, Maarten Jung, Jonathon Love, Fernando Miguez, Hannes Riebl, and Henrik Singmann. 2023. “Emmeans: Estimated Marginal Means, Aka Least-Squares Means.” https://cran.r-project.org/web/packages/emmeans/index.html.

Liang, Dan, Shengnan Yang, Emilio Pagani-Núñez, Chao He, Yang Liu, Eben Goodale, Wen Bo Liao, and Junhua Hu. 2020. “How to Become a Generalist Species? Individual Niche Variation Across Habitat Transformation Gradients.” *Frontiers in Ecology and Evolution* 8. https://www.frontiersin.org/articles/10.3389/fevo.2020.597450.

McGroddy, Megan E., Tanguy Daufresne, and Lars O. Hedin. 2004. “Scaling of C:N:P Stoichiometry in Forests Worldwide: Implications of Terrestrial Redfield-Type Ratios.” *Ecology* 85 (9): 2390–2401. https://doi.org/10.1890/03-0351.

Menke, Sean B., Benoit Guénard, Joseph O. Sexton, Michael D. Weiser, Robert R. Dunn, and Jules Silverman. 2011. “Urban Areas May Serve as Habitat and Corridors for Dry-Adapted, Heat Tolerant Species; an Example from Ants.” *Urban Ecosystems* 14 (2): 135–63. https://doi.org/10.1007/s11252-010-0150-7.

Moore, John C., Eric L. Berlow, David C. Coleman, Peter C. de Ruiter, Quan Dong, Alan Hastings, Nancy Collins Johnson, et al. 2004. “Detritus, Trophic Dynamics and Biodiversity.” *Ecology Letters* 7 (7): 584–600. https://doi.org/10.1111/j.1461-0248.2004.00606.x.

Oksanen, Jari, Roeland Kindt, Gavin L. Simpson, and Duncan Murdoch. 2023. “Vegan3d: Static and Dynamic 3D Plots for the ‘vegan’ Package.” R Package. R. https://cran.r-project.org/web/packages/vegan3d/index.html.

Oksanen, Jari, Gavin L. Simpson, F. Guillaume Blanchet, Roeland Kindt, Pierre Legendre, Peter R. Minchin, R. B. O’Hara, et al. 2022. “Vegan: Community Ecology Package.” R Package. R. https://cran.r-project.org/web/packages/vegan/index.html.

Ortiz-Sepulveda, Claudia M., Bert Van Bocxlaer, Andrés D. Meneses, and Fernando Fernández. 2019. “Molecular and Morphological Recognition of Species Boundaries in the Neglected Ant Genus Brachymyrmex (Hymenoptera: Formicidae): Toward a Taxonomic Revision.” *Organisms Diversity & Evolution* 19 (3): 447–542. https://doi.org/10.1007/s13127-019-00406-2.

Penick, Clint A., Amy M. Savage, and Robert R. Dunn. 2015. “Stable Isotopes Reveal Links between Human Food Inputs and Urban Ant Diets.” *Proceedings of the Royal Society B: Biological Sciences* 282 (1806): 20142608. https://doi.org/10.1098/rspb.2014.2608.

Posit team. 2023. “RStudio: Integrated Development Environment for R.” Boston, MA, USA: Posit Software, PBC. http://www.posit.co/.

R Core Team. 2022. “R: A Language and Environment for Statistical Computing.” R. Vienna, Austria: R Foundation for Statistical Computing. https://www.R-project.org/.

Raubenheimer, David, Steven J. Simpson, and David Mayntz. 2009. “Nutrition, Ecology and Nutritional Ecology: Toward an Integrated Framework.” *Functional Ecology* 23 (1): 4–16. https://doi.org/10.1111/j.1365-2435.2009.01522.x.

Santos, Marcus Nascimento, Jacques H. C. Delabie, and Jarbas M. Queiroz. 2019. “Biodiversity Conservation in Urban Parks: A Study of Ground-Dwelling Ants (Hymenoptera: Formicidae) in Rio de Janeiro City.” *Urban Ecosystems* 22 (5): 927–42. https://doi.org/10.1007/s11252-019-00872-8.

Smith, Ian A., Victoria K. Dearborn, and Lucy R. Hutyra. 2019. “Live Fast, Die Young: Accelerated Growth, Mortality, and Turnover in Street Trees.” *PLOS ONE* 14 (5): e0215846. https://doi.org/10.1371/journal.pone.0215846.

Takahashi, Maressa Q., Jessica M. Rothman, David Raubenheimer, and Marina Cords. 2019. “Dietary Generalists and Nutritional Specialists: Feeding Strategies of Adult Female Blue Monkeys (Cercopithecus Mitis) in the Kakamega Forest, Kenya.” *American Journal of Primatology* 81 (7): e23016. https://doi.org/10.1002/ajp.23016.

Terando, Adam J., Jennifer Costanza, Curtis Belyea, Robert R. Dunn, Alexa McKerrow, and Jaime A. Collazo. 2014. “The Southern Megalopolis: Using the Past to Predict the Future of Urban Sprawl in the Southeast U.S.” *PLOS ONE* 9 (7): e102261. https://doi.org/10.1371/journal.pone.0102261.

Theis, Kevin, Pierre Quévreux, and Michel Loreau. 2022. “Nutrient Cycling and Self-Regulation Determine Food Web Stability.” *Functional Ecology* 36 (1): 202–13. https://doi.org/10.1111/1365-2435.13961.

Ward, Philip S, and Bonnie B Blaimer. 2022. “Taxonomy in the Phylogenomic Era: Species Boundaries and Phylogenetic Relationships among North American Ants of the Crematogaster Scutellaris Group (Formicidae: Hymenoptera).” *Zoological Journal of the Linnean Society* 194 (3): 893–937. https://doi.org/10.1093/zoolinnean/zlab047.

Wei, Hongxu, and Xingyuan He. 2021. “Foliar C/N Stoichiometry in Urban Forest Trees on a Global Scale.” *Journal of Forestry Research* 32 (4): 1429–43. https://doi.org/10.1007/s11676-020-01188-6.

Wist, Bianca, Caroline Stolter, and Kathrin H Dausmann. 2022. “Sugar Addicted in the City: Impact of Urbanisation on Food Choice and Diet Composition of the Eurasian Red Squirrel (Sciurus Vulgaris).” *Journal of Urban Ecology* 8 (1): juac012. https://doi.org/10.1093/jue/juac012.

Yanoviak, S. P., and M. Kaspari. 2000. “Community Structure and the Habitat Templet: Ants in the Tropical Forest Canopy and Litter.” *Oikos* 89 (2): 259–66. https://doi.org/10.1034/j.1600-0706.2000.890206.x.

Youngsteadt, Elsa, Ryanna C. Henderson, Amy M. Savage, Andrew F. Ernst, Robert R. Dunn, and Steven D. Frank. 2015. “Habitat and Species Identity, Not Diversity, Predict the Extent of Refuse Consumption by Urban Arthropods.” *Global Change Biology* 21 (3): 1103–15. https://doi.org/10.1111/gcb.12791.
