# Supplementary material for: Urbanization strengthens vertical stratification of ant nutrient preferences in a temperate forest ecosystem
Source: PLoS One. 2025 Mar 28;20(3):e0320380. doi: 10.1371/journal.pone.0320380 (PMC11952225; doi:10.1371/journal.pone.0320380)
Supplement: Attachment — Submitted filename: response.docx [file pone.0320380.s003.docx]

PONE-D-24-15366

Urbanization strengthens vertical stratification of ant nutrient preferences in a temperate forest ecosystem

PLOS ONE

Additional Editor Comments:

Dear Dr. Kirchner,

After this first review round, all three reviewers believe your manuscript deserves to be accepted to be published in PLoS One after major/minor reviews.

In general, all three reviewers enjoyed reading your research, but they believe both structural (written English) and theoretical improvements could be implemented to your study. Please do not forget to prepare a rebuttal letter to the reviewers informing about all the changes you implemented and justifying tose you did not accept. Considering the amount of improvements, I will grant you a three-month period to deliver the new version of the study. Please do no hesitate to write me in case you need more time. Otherwise, you also do not have to hesitate to resubmit earlier than my established due in cse you can.

Sincerely,
Daniel Silva

Thank you for the positive feedback and the opportunity to revise our manuscript! We address all referees’ comments in detail below; **line numbers in the response refer to the tracked changes version of the manuscript**. In addition, we corrected the manuscript formatting to follow PLOS One’s style guides, added tables and figure captions into the manuscript text, submitted figures as individual files, included Supporting Information captions at the end of the manuscript, and included the full ethics statement in lines 121–127. We provide an amended funding statement below and in our cover letter.

MK was supported by a Research Assistantship with North Carolina State University Department of Entomology and Plant Pathology. The research was also supported by a North Carolina Wildlife Federation Scholarship Grant and a North Carolina Entomological Society Student Development Award to MK. The funding sources did not provide grant numbers. The funders played no role in the research or publication process. There was no additional external funding received for this study.

Reviewer's Responses to Questions

**Comments to the Author**

1. Is the manuscript technically sound, and do the data support the conclusions?

The manuscript must describe a technically sound piece of scientific research with data that supports the conclusions. Experiments must have been conducted rigorously, with appropriate controls, replication, and sample sizes. The conclusions must be drawn appropriately based on the data presented.

Reviewer #1: Yes

Reviewer #2: Yes

Reviewer #3: Partly

2. Has the statistical analysis been performed appropriately and rigorously?

Reviewer #1: Yes

Reviewer #2: Yes

Reviewer #3: Yes

3. Have the authors made all data underlying the findings in their manuscript fully available?

The [PLOS Data policy](http://www.plosone.org/static/policies.action#sharing) requires authors to make all data underlying the findings described in their manuscript fully available without restriction, with rare exception (please refer to the Data Availability Statement in the manuscript PDF file). The data should be provided as part of the manuscript or its supporting information, or deposited to a public repository. For example, in addition to summary statistics, the data points behind means, medians and variance measures should be available. If there are restrictions on publicly sharing data—e.g. participant privacy or use of data from a third party—those must be specified.

Reviewer #1: No

Reviewer #2: Yes

Reviewer #3: Yes

4. Is the manuscript presented in an intelligible fashion and written in standard English?

PLOS ONE does not copyedit accepted manuscripts, so the language in submitted articles must be clear, correct, and unambiguous. Any typographical or grammatical errors should be corrected at revision, so please note any specific errors here.

Reviewer #1: Yes

Reviewer #2: Yes

Reviewer #3: Yes

**Reviewer #1:** I liked it very much because it is very well written, easy to follow, well organized in terms of data analysis, and following results and discussion sections. The figures in the main text are also very well constructed. Another positive aspect is the scenarios (and analyses) and details on results regarding the ants related to each scenario. Further comments can be found below:

Thank you for your time and helpful comments on our manuscript!

- line 129: vouchers will be deposited? They should be prior to publication;

Thank you for pointing this out! This was a holdover from a previous draft when we hadn’t deposited the vouchers yet. I updated the language in line 141 of the main text to reflect that we have already deposited our vouchers in the museum. We also updated the language in Text S2 of the supplement.

- line 136: citing Rstudio should be ‘RStudio Team (2023). RStudio: Integrated Development for R. RStudio, PBC, Boston, MA URL [http://www.rstudio.com/.](http://www.rstudio.com/)’

RStudio Inc. changed its name to Posit in 2022. If you follow the link to rstudio.com, you are now redirected to posit.co. Running RStudio.Version() in the most recent version of RStudio gives the following citation information:

```
To cite RStudio in publications use:

  Posit team (2024). RStudio: Integrated Development Environment for R.
  Posit Software, PBC, Boston, MA. URL http://www.posit.co/.
```

- line 144: please, explain in more detail which means the ‘additive effect of site’;

Here we are distinguishing between the effects of stratum and habitat type which are allowed to interact with each other and the effect of site which is not. We added clarification in line 160 that the effect of site is not interactive. In other models, site was included as a random effect, but because the “adonis2” function, which we used to perform the PERMANOVA, does not allow for a random effect structure, we included site as a fixed nuisance variable in this analysis. This way, the PERMANOVA results still account for the variability between sites and more accurately represent our sampling design.

- line 171: checked diagnostic plots for the mixed model should be included in supplementary material;

It is our understanding of the PLOSOne author instructions that including the model diagnostic plots in the supplement would not be required, because the raw data and code needed to produce the models are readily available. Following this comment would result in adding at least 31 plots to the supplement given the number of models included in this manuscript and the number of diagnostic plots per model. We would prefer to not weigh down the supplement with so many plots, but we will be happy to do so if the editor indicates that this is, in fact, required.

- line 194: tropical species? It’s possible understand that in lines 260-261; needing clarify this at the first approach;

We clarified in lines 224–225 and line 331 that the tropical species we’re referring to are non-native.

- line 205-208: would be important to show the average number of species by tree (and sd) to understand the diversity dimension in trees;
We do not consider tree to be our lowest level of replication in this study. Because trees within a site are not independent sampling units, analyses at the level of tree without appropriate random effects structure would represent pseudoreplication. The rarefaction is performed at the level of site, which is why we report habitat-level estimates of diversity. Any reports of tree-level diversity would be simple summary statistics rather than model outputs. However, to satisfy another reviewer request, we have included the number of trees in which each species was collected (in addition to the number of sites, i.e., the number of independent replicates) in Table S4. We have also added a sentence at the beginning of our data analysis section (lines 148–151) to clarify that site is our level of replication.

- line 207: please, clarify meaning of ‘urban arboreal, forest arboreal, combined with numbers [forest terrestrial: 2’]

We added context in lines 240–241 for clarification.

- line 280-281: should also add that baiting does suffer of dominance effect in ant sampling;

We added a nod to dominance effects in lines 352–353 of the discussion.

- line 291-292: this is a central find in the study; on the other hand, would be expected because canopy structure in tropical forest is much more compartmentalized than temperate canopy (taller trees, without branches near to the ground, large biomass of epiphytic vegetation, etc); perhaps this should be added because there are huge differences between the two systems (temperate vs. tropical trees);

We elaborated on specific differences between temperate and tropical forests in lines 67–71 of the introduction.

- line 333-334: that is strange – such information did not appear in the M&M section, but it is showed in almost last message of the study;
In lines 134–136 of the Methods section, we report the specific foods we used for baits and refer to Table S1, which contains a complete breakdown of the nutritional content of these foods. This information was included in the original submission; to further clarify, we now emphasize in line 404 that we’re referring to the tuna and jam baits we used in this study.

- Table S4: row number 34 is composed by two species names.

Good catch! We added an explanation footnote to the table caption: “**Nylanderia vividula* and *N. terricola* workers cannot be reliably separated based on morphology, so we treated these two species as one for this study.”

**Reviewer #2:** Find below my comments and suggestions about the study. I found it very interesting and relevant. You present a very robust and well-constructed design and data analysis with the corresponding interpretations. My only concern in a few analyses is about you may be not taking into consideration the dependence of sampling from the same trap. We know that the abundance of workers (and hence biomass) may be related to factors such as proximity to the nest or other sources of food. In this sense, there is a dependency on the biomass of ants of one species in one bait with the biomass on the other bait of the same trap (see below). Perhaps I am wrong, but I suspect that considering the absolute biomass or not using the trap as a random variable may risk misleading conclusions, at least theoretically.

Thank you for your detailed review of our manuscript! We are aware of the potential non-independence of baits at the same trap and address these concerns in more detail below.

Detailed comments
• Line 72-74: I agree with your general statement that differences in vertical structure may translate into differences in animal preferences. However, you should be more specific about what those differences are between forest types (tropical vs temperate) to better highlight the consequential effects on animals.

We elaborated on specific differences between temperate and tropical forests in lines 67–71.

• Lines 95-102: The main hypothesis and predictions are well introduced. They concern two main response variables: ant diversity and food preference, in relation to two main predictors: strata and urbanization level. Nevertheless, for the first response (diversity), you presented expectations for predictors only as independent variables, while for the second response (preference), the expectation implies an interaction between predictors. As many of the further analyses consider the interaction among predictors, I suggest mentioning the expectations about the interaction for ant diversity and possible mechanisms behind it.

Thank you for pointing out this oversight. We clarified our wording in lines 93–96 to specify the interaction for the diversity predictions as well.

• Lines 112-113: Perhaps you could describe (at least qualitatively) the general structure of the vegetation (in both forest and urban sites), specifically the tree height and vegetation density.

We added a short description to lines 117–120. We added a reference to Fig. 1a which includes a visual of a typical forest and typical urban site. We now also address bait height in Text S3 of the supplement.

• Lines 113-117: As community richness may be dependent on habitat area size, you should provide the sizes of sampling areas or at least some estimation of their variability. Could this be responsible for some of the observed variation?

We attempted to keep sampling area and effort as comparable as possible between the urban and forest habitats. We added additional information to lines 117–120 to address this comment.

• Lines 121-122: I found this bait station design very interesting and promising. It may help address nutrient preferences in other habitats. However, I think you should mention (or test, if possible) how (or if) dominance relationships among ant species on bait finding may introduce bias in the results. Also, consider that this design introduces a degree of dependency of samples with different baits from the same trap, which should be considered in the data analysis.

We added a mention to dominance relationships at baits in lines 325–353 of the discussion. We address the issue of non-independence in detail under the comment for Line 170.

Out of curiosity, we checked and in ~1/3 of our baits which contained ants, we collected more than one species!

• Line 127: Were samples from different exposure times (4h and 8h) taken as independent samples or pooled together? Also, were baits replaced after 4h removal? Otherwise, could any chemical signals affect subsequent samples? (after reading the results I understood that this data were pooled together, you should mention this here)

We would argue that pooling data is a data analysis method, not a sampling method, and the explanation of pooling data fits better in lines 177–180 of the methods than in this paragraph.
We did, however, add a statement that baits were completely replaced after 4h to lines 139–140.

• Lines 131-133: Were ants from the same species weighed pooled together (and a mean value calculated) or individually?

We added clarification to lines 145–146. We weighed every unique combination of bait and ant species. Ants of the same species from the same bait were dried and weighed together, and all baits were weighed separately. So if we had species A and species B in bait 1 and species A in bait 2, we dried and weighed all of species A from bait 1 separately from species B in bait 1 and species A in bait 2.

See lines 177–186 for how we calculated biomass estimates.

• Lines 141-142: You should explain the purpose of removing singletons.

We added clarification to line 157.

• Lines 152-153: Perhaps I missed it, but I couldn’t find the results of betadisper neither in the results nor in the interpretation in the discussion.

See lines 260–262 of the results.

• Lines 156-158: Was there any reason for not performing the same beta partition procedure among urban and forest communities?

It was just not a question we had planned on asking! We have now included this analysis and accordingly updated the methods in lines 171–175, added the results to lines 266–269, and added Figure S4 to the supplement.

• Line 160: I calculated the total number of trees following the description of the sampling method, reaching 84 trees in total. Am I right? If so, what happened to the missing tree? (You explain this later, but putting 83 at this point of the text is confusing.)

Fixed! In lines 177–178, we revised to refer to ‘each sampled tree’ rather than a specific number of trees.

• Line 166: You should report the description year along with the author for scientific names.

We report the description year for each species in Table S4. PLOSOne author instructions do not require the authority or year in the text.

• Line 170-171: Did you consider including the trap as a random variable? I think it is very important to account for dependency issues among baits of the same trap and hence the comparison among strata.

Yes, we have considered the non-independence of baits at the same bait station, but the model we present in the manuscript (which pools across traps within a tree, henceforth the ‘original model’) presents qualitatively the same results, is a better fit for the data, and is quantitatively more conservative than the model that includes bait-level results with bait station (trap) as a random effect (henceforth the ‘bait-level model’).

When we pooled the biomass data at the level of individual trap, i.e., the only collections pooled are the 4hr and 8hr collections of the same nutrient from the same bait station, and ran the model with a random effect of bait station nested in tree, the results are nearly identical, except for the following:

- The bait-level model is a worse fit to the data, violating the normality assumption. Likely this level of violation wouldn’t affect the validity of the model, but it is worth noting.

- The random and residual variance (of course)

Original model:

```
Random effects:
 Groups    Name        Variance Std.Dev.
 tree_code (Intercept) 0.5008   0.7077
 site           (Intercept) 0.3163   0.5624
 Residual                    1.8759   1.3696
Number of obs: 332, groups:  tree_code, 83; site, 14
```

Bait-level model:

```
Random effects:
 Groups          Name        Variance Std.Dev.
 station_code (Intercept) 0.2923   0.5406
 tree_code     (Intercept) 0.3376   0.5810
 site               (Intercept) 0.2149   0.4636
 Residual                   1.6575   1.2874
Number of obs: 662, groups:  station_code, 331; tree_code, 83; site, 14
```

- The biomass estimates are halved. This is to be expected, because in the original model, the biomass estimates pooled 4 collection instances (4hr and 8hr from the same trap AND the 2 traps in each tree), whereas the bait-level model pools only 2 collection instances (4hr and 8hr from the same trap). But as you can see the overall relative patterns are nearly identical to Fig. 3.

- Finally, the urban terrestrial carbohydrate preference is now significant in the bait-level model, even after adjusting for multiple comparisons (p=0.002). In the original model, the preference for carbs on the urban ground was not statistically significant after correcting for multiple comparisons (p=0.08).

Given these differences, we are inclined to keep the original model, because it fits the data better and is more conservative. We added a sentence in lines 179–180 to emphasize that pooling over bait station is still a relatively small amount of pooling overall (4 collection instances).

• Lines 187-188: Was there a reason for not using the same package (glmmTMB) in the previous GLMMs? If possible, use the same; the results should be equal or very similar, but you’ll maintain consistency in estimation.

We had to use glmmTMB for the beta regressions, because lme4 does not support the beta family distributions. However, when we started these analyses, glmmTMB was not supported by DHARMa, and so the model diagnostics would have been less reliable than those available for lme4. Thus, we preferred to use lme4 whenever possible. Very recently, it seems DHARMa has updated to support model diagnostics for glmmTMB, which is great news!

In this case, though, we do not think it is necessary to re-analyze everything in glmmTMB, because the estimation procedures between a linear mixed model and a generalized linear mixed model are not directly comparable, as they rely on different distributions. It seems more critical to us that the estimation procedures of all our linear mixed models are comparable (in this case in lme4) and that those of our generalized linear mixed models are also directly comparable (in this case in glmmTMB).

• Lines 190-192: I think you should better justify your criteria to define dominant species. While total biomass alone may be a relevant factor, it may underestimate the importance of small but aggressive species or even those with high recruiting capacity.

We are not using dominant in the sense of behavioral dominance (i.e., aggression vs. submission), but rather in the manner that we defined it on line 201, referring only to a species’ prevalence in the community biomass. We revised line 209 to state “biomass-dominant” to avoid confusion.

• Lines 192-193: This network approach seems interesting, but I found it confusing when trying to understand how it helps to clarify scenario b. It appears somewhat subjective (here you state that you visually assess the networks, but you later calculated some metrics) and fails to consider the particularity of your sampling design. Specifically, it suggests that all biomass measures (ground protein, ground carb, arboreal protein, and arboreal carb) are independent and directly comparable. Are they?

Scenario b was that the urban-forest differences in nutrient use arose from the altered behavior of one or a few massive taxa that dominated the community-wide result. The network visualization helps assess this scenario by illustrating, in each habitat, which taxa maintained the same nutrient-use patterns across habitats, and which ones shifted their patterns. We agree that this approach involves interpreting a visualization, not a statistical analysis, and we have removed the calculated connectance metrics to avoid confusion. We don’t believe that the network approach suggests independence, because in any bipartite network, organisms are choosing what or whom to interact with based on preference and/or competition. However, we do believe that the biomass measures between the strata and habitats are directly comparable because they are based on equal sampling effort and data pooling.

Additionally, we have updated our methods (lines 210–222) and results for scenario b (lines 298–313) to include statistical analyses for each biomass-dominant species in support of our network visualization.

• Lines 200-201: Perhaps you can also report the number (or even better the proportion) of baits occupied by ants in the urban and forest sites.

We added the proportion of baits occupied by ants in each habitat and stratum in lines 233–234.

• Lines 220-221: Was there a reason to use a 3D NMDS instead of a 2D NMDS to represent composition differences among habitat types? A 2D representation would be more easily interpreted, and I would prefer it as long as the stress remains somewhat low.

We determined the stress was too high (>0.2) for a 2D NMDS.

• Lines 240-241: I disagree with this interpretation. Connectance values seem to be very close to say safely that they are indeed different. You may provide a more objective evaluation, perhaps a null model approach.
We have removed the connectance metrics from the manuscript, as we don’t feel it adds very much to the interpretation of the results.

• Lines 241-254: I think you may support these statements about main dominant species with some estimation of effect size for individual species, such as estimated standardized coefficients from mixed models.

Thanks for the suggestion! We have now tested for behavioral changes between habitats and strata separately for each of the four biomass-dominant species with a linear mixed model. The results support our conclusions from our visual inspection. We have updated the methods (lines 210–222) and results (lines 298–313), and we added a supplemental table of model results (Table S7).

• Lines 249-252: You should report biomass estimates from habitats for both C. pennsylvanicus and F. subsericea. Perhaps it could be even better to include a table with biomass by species in the appendix.

We added Table S7 to the supplement which reports the raw biomass estimates for the four biomass-dominant species in each habitat and stratum.

• Lines 295-299: Your conclusion about differences in ant fauna composition is based on presence/absence data. If you compare the communities based on abundance or biomass-weighted matrices, do you think that it may better reflect the role of cross-habitat dominant species on community structure?

It is possible that using abundance-based matrices for the diversity analyses would change our results, however, such analyses would conflate community diversity/structure with behavior. Abundance measurements of ants are highly dependent on nest proximity and species-specific foraging and recruitment strategies, such that more individuals of the same ant species at a bait does not necessarily indicate more reproductive colonies in the community. We use biomass for our resource use questions, because it gives us an insight into how the community interacts within its environment, but for our diversity questions, we’re focused on community structure, not community dynamics. Gotelli et al. 2011 expands on why occurrence data is preferred for biodiversity analyses of social insects.

Gotelli, N.J., Ellison, A.M., Dunn, R.R. & Sanders, N.J. (2011) Biodiversity Sampling and Statistical Analysis for Myrmecologists. *Myrmecological News*, **15**, 13–19.

• Line 341: Do you mean the majority of species or individuals?

We mean the majority of species and biomass. We detected ~50% of species in both strata, and those species made up ~98% of biomass. We clarify in line 412 of the conclusion.

Finally, during the results, you highlight the different patterns derived from tropical invasive species. However, this topic is not treated in more depth in the discussion.

We added a sentence in lines 385–386 of the discussion to specifically mention the tropical non-native species.

**Reviewer #3:** In this manuscript, Michelle Kirchner, Elsa Youngsteadt and collaborators studied the effects of urbanization on a temperate forest ant community, regarding both vertical stratification (arboreal vs. ground) and macronutrients preference (carbohydrates vs. proteins). For doing this they compared seven sites in two US cities (Raleigh and Durham; North Carolina State) against seven sites in protected, secondary mixed hardwood forests in state parks, game lands, or private properties. At each site, they selected three trees of two species each (Quercus alba L. and Acer rubrum L.) and installed baits in the canopy and on the ground (two baits per strata at each tree). They present a novel bait station, consisting of a pine dowel with two styrene tubes attached to both ends, each containing a distinct bait type (either a carbohydrate-rich food, strawberry preserves, or a protein-rich food, tuna) and bearing drilled entry holes for guaranteeing ant access to the baits (see Supplementary material, Text S1, for more details). Their predictions were that (1) ants foraging in the canopy will prefer protein, while those on the ground will prefer carbohydrates; and (2) urbanization would eliminate nutrient preferences on the ground, but nutrient preferences in the canopy would remain unchanged.

Putting together, their results show that:
1) Ant community in both strata (ground and canopy) moderately overlapped in terms of species diversity and composition, both in urban and forest sites, what differs from tropical studies (such as Yanoviak and Kaspari 2000).
2) Although there was no nutrient*strata variation in ant preference regarding the forest habitat, the authors found that in the urban habitat, ants foraging on the canopy significantly preferred protein baits, whereas carbohydrates were almost significantly preferred in the ground. What was found for the urban habitat matches patterns described by tropical studies.

In spite of its merits, in its present form the manuscript needs to pass through moderate to major revisions in order to bring out the best of what has been discovered. My two major points of concern are the following:

1) Canopy height variation (see Page 6, Lines 127-129: “At each tree, we placed four bait stations, two in the canopy (mean height: 14.6m, range: 2.1–31.1m) and two on the ground (5m N and S of the base of the tree) (Fig. 1c) between 9:00 and 11:30am)”. – There is a huge difference between minimum (2.1 m) and maximum (31.1 m) values, and we are not informed about the standard deviation of their sampling heights. Note that if SD is high, we are probably dealing with heights that are very different to compare. While I do expect that ants foraging at 3 m height could easily forage on the ground, I do not expect that ants foraging at 25 m or more would come to the ground to forage. I am also asking myself if there is a specific bias in tree height related to the habitat: for example, that urban trees present smaller heights than forest tress. Therefore, authors should inform about sampling height for each habitat separately (mean and standard deviation). Moreover, they should discuss in which extent this variation could have affected their results (for example in the fourth paragraph of the discussion). Comparing with other studies cited by the authors, we are able to see that they present a smaller canopy height variance:
a. Yanoviak and Kaspari (2000, Oikos): canopy sampling occurred between 17 and 35 m above the ground;
b. Antoniazzi et al. (2020, Sociobiology): baits were installed at 10-15 m as high in the canopy as possible (10-15 m above the ground, depending on tree height).

Thank you for bringing this to our attention! We agree that our overall range is large, but that is primarily due to those two more extreme cases. It was an oversight that we failed to include the standard deviation (5.9m), which has been added to line 137.

To reassure the reader that bait height did not affect our results, we have now run a linear mixed model with bait height as a function of habitat and tree species. Baits were higher in oak trees and in the forest, but the height difference between species was equal in urban and forest sites. If the greater height of forest baits made them less accessible to cross-stratum foragers, we would expect certain patterns to appear in our data, which we did not find. We added Text S3 to the supplement detailing these considerations. Additionally, we added Fig. S1 which plots the estimated marginal means, their confidence intervals, and the raw bait heights for each habitat and tree species, so the reader can better visualize the bait height variation.

Specific to this concern: “I do not expect that ants foraging at 25 m or more would come to the ground to forage”. We also have data (in revision in another manuscript) from climbing 18-24m into these same forest canopies and hand collecting ants. During those collections, we watched ants climb from their nests on the ground up into the canopy to forage above our heads. We agree that arboreal specialists likely would not make the journey down—even from lower points in the canopy. Arboreal specialists also make up a minor share of our total ant diversity (~12%). So, we are reasonably confident that the average 6.6m difference between forest and urban baits is not a factor of concern for our cross-stratum foragers.

2) In the discussion, I missed any mention to the three tropical invasive ants that were found in this study (Brachymyrmex patagonicus Mayr, Linepithema humile (Mayr), and Solenopsis invicta). Even though, in the results, Solenopis invicta is mentioned as being more abundant in the city (forest biomass: 0.6mg, urban biomass: 838.5mg) than in the forest, and forages equally on carbohydrates and protein in both strata (see page 12, lines 259-260). Based on Table S4, we can see that these three species seem to be slightly more common in the terrestrial than in the arboreal community. Does this fat present any consequence to the patterns discussed for urban areas?
We added a sentence in lines 385–386 of the discussion to specifically mention the lack of effect of tropical non-native species on the observed preference shift. We specifically mention *S. invicta* in the results, because, even though it is more abundant in the city and makes up >10% of the urban ant biomass, it does not prefer a specific nutrient. Its biomass is split equally between carbohydrate and protein baits in both strata, so it is not driving the pattern of carbohydrate preference on the ground or protein preference in the canopy. The other two tropical, non-native species, *Brachymyrmex patagonicus* and *Linepithema humile*, exhibit the same behavior and they make up a very small share of the overall urban biomass (species 6 and 28 on Fig. 4). We don’t think it is surprising that these species were collected more often on the ground than in the canopy, because they are all known ground-nesters. Even still, when they *are* present in the canopy, they still exhibit no nutrient preference, as detailed in the final paragraph of the results section.

MINOR COMMENTS

WRITING: Despite the fact that I am not a native English speaker, and that the authors are native speakers, I have made a few suggestions about words changing as well as sentences organization. These suggestions are included directly in the revised manuscript attached. Note that they are not mandatory.
Thank you for the suggestions. We have incorporated some of these into the manuscript.

KEYWORDS: I have made some suggestions regarding keywords choice directly in the revised manuscript.

Upon revision, we realized that PLOSOne does not use keywords, and so these have been removed altogether.

ABOUT THE TREE-SPECIES USED: two tree species (white oak, Quercus alba L.; and red maple, Acer rubrum L.) were used with equal numbers (three trees per site) in both habitats, thus potentially eliminating differences in ant attendance that could emerge from plant species. However, the authors did not mention if both species commonly present ant-attractants, either sap-feeders hemipterans of the family Membracidae (Hemiptera) or extra-floral nectaries. Moreover, even though both are common species in the US, it is important to mention their family names, since Plos One audience is worldwide.

We added a sentence to lines 113–114 to clarify that both species host honeydew-producing insects, and we added the family names for the tree species!
I also missed any mention in the results to the identity of tree species: did this fact affect ant composition and diversity?

We did not test for this specifically with this dataset, but we have another manuscript in revision which shows that ant diversity & composition is similar amongst these tree species.

VOUCHERS DEPOSITION: in page 6 (lines 129-130), as well as on the Appendix with supporting information (see “Text S2: Description of Tapinoma cf. sessile”), the authors state that “Vouchers will be deposited in the North Carolina State University Insect Museum.” Normally, when research is published, vouchers are already deposited in a collection/museum and the authors mention specimens IDs for enabling readers finding the deposited specimens.

Thank you for pointing this out! This was a holdover from a previous draft when we hadn’t deposited the vouchers yet. I updated the language in line 141 and Text S2 to reflect that we have already deposited our vouchers in the museum. We have refrained from providing specific IDs because we submitted >1200 vouchers.

RESULTS: Page 9 (lines 199-201) - I would suggest the authors to include the total number of baiting possibilities. Following my calculations, there were 1344 baiting possibilities and having retrieved a total of 1321 is very expressive.

Fixed! In line 230, we added the total number of possible baits.

LITERATURE USAGE: I noticed that the authors included many relevant and new references (from 2020 ahead). Nonetheless, I would like to advise the authors to search for the following recent works, which may be interesting to include:
- Zuanon, L.A.; Leão, R.E.O.S.; Quero, A.; Neves, K.C.; Vasconcelos, H.L. Nutrient Supplementation to Arboreal Ants: Effects on Trophic Position, Thermal Tolerance, Community Structure and the Interaction with the Host-Tree. Diversity 2023, 15, 786.
- Camarota, F., Vasconcelos, H. L., Koch, E. B., & Powell, S. (2018). Discovery and defense define the social foraging strategy of Neotropical arboreal ants. Behavioral Ecology and Sociobiology, 72, 1-11.
Thank you! We included a reference to the Zuanon et al. paper which supports our explanation for higher *Crematogaster ashmeadi* biomass in the urban habitat in lines 401–403.

FIGURE CAPTIONS: figure captions could not be corrected directly on the Word file. So, here are some observations:
Figure 1 – Instead of “cartoon” use the more adequate term “illustration”.

Fixed!

Figure 2 – I would rather suggest the authors to show the two rarefaction curves of each habitat type (forest and urban) together in a unique figure, making it easier to compare the species richness among the four combination (strata and habitat). Note that when this figure is referred in the manuscript (see page 10, lines 214-216), the four curves are compared.

The curves are intentionally separated by habitat so that the confidence intervals are clearly separated. If all 4 curves were on the same plot, the CIs of each curve would be difficult to distinguish from one another. The four curves all share the same scale, merely the origin for the urban plots is shifted to the right.

Figure 4 – Please, use the more informative term “Bipartite Interaction networks” instead of “network” alone.

Fixed!

Figure 4 (lines 28 -31): “Nutrients for which we observed a preference shift across habitat types are highlighted. Species that make up a large portion of community biomass and/or show a behavioral shift across habitat types are highlighted and named.” – I was not able to perceive how nutrientes were highlighted in the networks, I only noted that some ant species were highlighted.

The lines connecting species to arboreal protein are highlighted with a tan color (matching figure 3), and the lines connecting species to terrestrial carbohydrates are highlighted with a pink color (matching figure 3). These are the two stratum*nutrient combinations for which we see a shift in preference from the forest to the urban community. The remaining 2 stratum*nutrient combinations are left grey. We added this distinction to the caption.

REFERENCES FORMATTING:
- The authors did not follow Plos One guidelines for references, which state the following “References are listed at the end of the manuscript and numbered in the order that they appear in the text. In the text, cite the reference number in square brackets (e.g., “We used the techniques developed by our colleagues [19] to analyze the data”). PLOS uses the numbered citation (citation-sequence) method and first six authors, et al.” . Here, the authors used names and year for citing references throughout the text.
- Moreover, the reference list is, neither numbered (as mentioned above), nor formatted as indicated in the guidelines “PLOS uses the reference style outlined by the International Committee of Medical Journal Editors (ICMJE), also referred to as the “Vancouver” style”.
- In this sense, authors should format the references of the entire manuscript.
- Please, also note that species names in the titles of some references are not italicized.

All formatting has been fixed.

SUPPLEMENTARY MATERIAL:
- Table S4 shows all species collected: They are sorted alphabetically and no mention to their subfamilies is made. I would recommend the authors to include subfamily names and organize species by subfamily and then inside each subfamily, alphabetically. I would also suggest the authors to show in this table the effective number of trees (or baits) harboring each ant species. At the present version, authors show only the number of sites (maximum is seven for each habitat) where each ant was found.

We have added subfamily information to Table S4, but we have left it ordered alphabetically, because it is used as the legend for the numbered species of Fig. 4. Reordering the table by subfamily would make it more difficult for the reader to navigate Fig. 4.

We do not consider tree or bait to be our lowest level of replication in this study, which is why we include site-level occurrences for each species. We have added a sentence at the beginning of our data analysis section (lines 148–151) to clarify that site is our level of replication. However, to satisfy this (and another reviewer’s) comment, we have added the number of trees (across all 7 sites) in which each species was present in parentheses to Table S4.

- The list of supporting materials (text, Tables and figures) is not shown at the end of the manuscript, as stated in Plos One guidelines: “List supporting information captions at the end of the manuscript file. Do not submit captions in a separate file.”
- Authors should also avoid mentioning “Appendix S1” everytime on the text that they want to refer to any supplementary material. It is better to only mention the specific code/number of this material. Instead of “(Appendix S1: Text S2)”, just mention “(Text S2)”.

All formatting has been fixed.

- I would like to suggest authors to improve almost all captions for the supplementary material in order to give the potential readers more details of the study, which otherwise are found only in the manuscript file. By finding more information in the captions, readers will understand/remember study details better.

We added context to almost all of the tables and figures in the supplement!

Note that some other small comments regarding methodology, references, and others were also included directly on the revised manuscript file.

We included responses to some of the comments in the tracked changes version of the revised manuscript.
