# Supplementary material for: Urbanization strengthens vertical stratification of ant nutrient preferences in a temperate forest ecosystem
Source: PLoS One. 2025 Mar 28;20(3):e0320380. doi: 10.1371/journal.pone.0320380 (PMC11952225; doi:10.1371/journal.pone.0320380)
Supplement: Attachment — Submitted filename: response_auresp_2.docx [file pone.0320380.s005.docx]

PONE-D-24-15366

Urbanization strengthens vertical stratification of ant nutrient preferences in a temperate forest ecosystem

PLOS ONE

Comments to the Author:

**Reviewer #2:** Dear Authors,

After carefully considering your explanations and the revisions made to the manuscript, I am pleased with the adjustments, particularly regarding the concerns I had about the statistical models used in the previous version. I find your responses satisfactory and appreciate the effort you put into addressing these points.

It may still be worth mentioning briefly—perhaps even in the supplementary material—how you handled the potential issue of non-independence of baits, as you explained well in your response. Including this could provide helpful context for future readers but it is not a condition to aceptance.

In light of these improvements, I am happy to recommend the acceptance of your manuscript.

Best regards,

Thank you for your time and comments! We agree and added a short explanation of our decision to use the pooled model to lines 186–190.

**Reviewer #3:** In this new version of their manuscript, Michelle Kirchner, Elsa Youngsteadt and collaborators apparently addressed and solved most of the points raised by me and the other two reviewers.

In the previous version, my principal concern was on how bait height was distributed between urban and forest sites, as well as if bait height was too low for not allowing stratification of ants between canopy and ground strata. This concern was addressed and clarified by the authors, with some inserts in the manuscript itself as well as the addition of some supporting material (Text S3, andd Figure S1).

Thank you for your time and comments!

Manuscript formatting was also thoroughly revised by authors. However, I feel that figure captions and tables are still not entirely correct. According to the model given by Plos One, they should be inserted on the text separated by a blank line

In [PLOS ONE’s sample manuscript body](https://journals.plos.org/plosone/s/file?id=9cba/PLOS%20Manuscript%20Body%20Formatting%20Guidelines.pdf), the spacing between sections and captions is inconsistent, likely due to the annotations. For example, there is no blank line between the end of the “Introduction” and the heading of the next “Header 1” section, but there are blank lines before "Introduction” header. As for captions, the first figure caption is preceded by a blank line, but the second figure caption is not. Neither the table caption nor footer is not preceded by a blank line. Given these inconsistencies, we have left the formatting as is, i.e., with no additional blank lines, and we hope the editorial staff can provide clarification during the final checks.

I attached a revised version of the manuscript with very few (and small) corrections.

We incorporated some of the wording changes into the manuscript. We address specific comments in the manuscript below:

We are not sure what changes the reviewer intended with respect to our Table 1 formatting. We have double checked the PLOS ONE sample manuscript body and the table formatting instructions, and we believe our table complies. Again, hopefully editorial staff will provide clarity on final checks.

In line 102 of the Methods section, we refer to Raleigh-Durham as a “major metropolitan area”, and thus, we have not changed the mention of an “urban metropolis” in the first sentence of the Discussion (line 330).

We don’t wish to distract from the specific discussion of our results with the introduction of a new hypothesis so late in the paper. In lines 389–391, we are suggesting a potential alternative mechanism for the patterns we observed. The mechanism we suggest would be supported by the findings in Kaspari et al. 2012, and so, we want to reference that paper. However, we were concerned that an unannotated reference to Kaspari et al. 2012 could imply that their results apply specifically to our suggested case, i.e., that they have results from urban landscapes with anthropogenic carbohydrate inputs. We included the specific mention of the economics hypothesis to clarify for the reader the specific portion of the paper to which we’re referring. We have adjusted our citation to make this intention clearer; it now reads “see the economics hypothesis in 2”, where 2 refers to Kaspari et al. 2012.
